# An Emerging Liquid-Crystalline Conducting Polymer Thermoelectrics: Opportunities and Challenges

**DOI:** 10.1007/s40820-025-01916-9

**Published:** 2025-11-15

**Authors:** Zhenqiang Ye, Mingdong Zhang, Junyang Deng, Lirong Liang, Chunyu Du, Guangming Chen

**Affiliations:** College of Materials Science and Engineering, Shenzhen University, Shenzhen, 518055 People’s Republic of China

**Keywords:** Thermoelectric materials, Polymer, PBTTT, Liquid-crystalline

## Abstract

Thermoelectric (TE) materials, being capable of converting waste heat into electricity, are pivotal for sustainable energy solutions. Among emerging TE materials, organic TE materials, particularly conjugated polymers, are gaining prominence due to their unique combination of mechanical flexibility, environmental compatibility, and solution-processable fabrication. A notable candidate in this field is poly(2,5-bis(3-alkylthiophen-2-yl)thieno[3,2-b]thiophene) (PBTTT), a liquid-crystalline conjugated polymer, with high charge carrier mobility and adaptability to melt-processing techniques. Recent advancements have propelled PBTTT’s figure of merit from below 0.1 to a remarkable 1.28 at 368 K, showcasing its potential for practical applications. This review systematically examines strategies to enhance PBTTT’s TE performance through doping (solution, vapor, and anion exchange doping), composite engineering, and aggregation state controlling. Recent key breakthroughs include ion exchange doping for stable charge modulation, multi-heterojunction architectures reducing thermal conductivity, and proton-coupled electron transfer doping for precise Fermi-level tuning. Despite great progress, challenges still persist in enhancing TE conversion efficiency, balancing or decoupling electrical conductivity, Seebeck coefficient and thermal conductivity, and leveraging melt-processing scalability of PBTTT. By bridging fundamental insights with applied research, this work provides a roadmap for advancing PBTTT-based TE materials toward efficient energy harvesting and wearable electronics.

## Highlights

- Poly(2,5-bis(3-alkylthiophen-2-yl)thieno[3,2-b]thiophene) (PBTTT) synthesis and main strategies to enhance its thermoelectric performance (including doping, composite engineering and aggregation state controlling) are comprehensively reviewed.
- The thermoelectric performances of PBTTT-related materials are systematically summarized and compared.
- Future opportunities of PBTTT thermoelectric performance enhancement and effective utilization of its unique melt processibility in multiscale regulation, composite and hybrid, and processing technology innovation are outlooked.

## Graphical abstract

**Figure:**
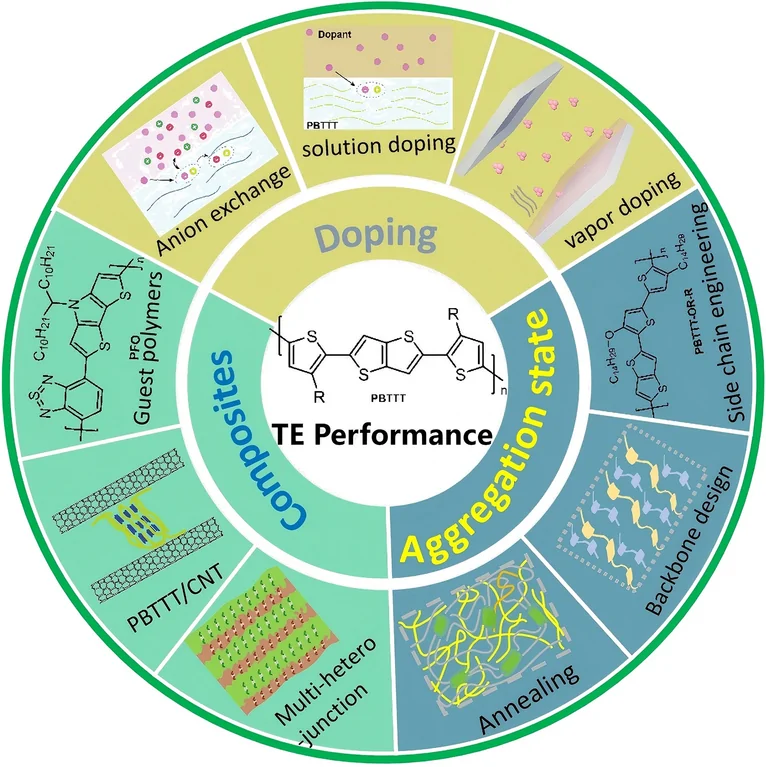

## Introduction

With growing global focus on sustainable development and carbon emission reduction, research on energy recovery and re-utilization has witnessed explosive advances [1]. Based on the Seebeck effect, a temperature difference across a material generates an electric potential by driving charge carriers from hot to cold regions, thermoelectric (TE) technology [2, 3] can directly and effectively convert heat to electrical energy and vice versa, thereby being effective in the recovery of waste heat and low-grade heat and displaying promising prospects in achieving low-carbon economy. The development of high-performance TE materials is vital. Historically, inorganic TE materials, such as bismuth telluride (Bi_2_Te_3_) [4], lead telluride (PbTe) [5] and tin selenide (SnSe) [6], have been extensively investigated. However, their disadvantages, including toxicity, low content in earth, high intrinsic rigidity and inevitable elevated energy consumption during processing, seriously limit their large-scale applications [7, 8]. Fortunately, the recently-developed organic counterparts [9, 10] reveal distinct advantages, covering low cost, exceptional flexibility [11, 12], facile processing [13–16], and eco-friendly non-toxicity [17, 18], which have garnered much of current interest. Notably, they have enabled diverse applications including artificial intelligence [19, 20], fire recognition and alarming [21–23], smart buildings [24, 25], wearable and flexible electronics [26–28]. So far, conjugated polymers [29, 30] represent the most extensively studied class of organic TE (OTE), being characteristic of their intrinsic conjugated π-electron systems that confer remarkable electronic properties [31–33]. The development of conjugated polymers can be traced back to 1977 [34]. Polyacetylene (PA) belongs to the first generation, possessing ultra-high conductivity [35]. However, air instability and processability limitations hindered its development. Subsequently, various conjugated polymers have been reported, such as polythiophene (PTh) [36, 37], polyaniline (PANI) [38, 39], polypyrrole (PPy) [40, 41], poly(3-hexylthiophene) (P3HT) [42, 43], and poly-(3,4-ethylene dioxythiophene) (PEDOT) [44–47].

**Fig. 1:**
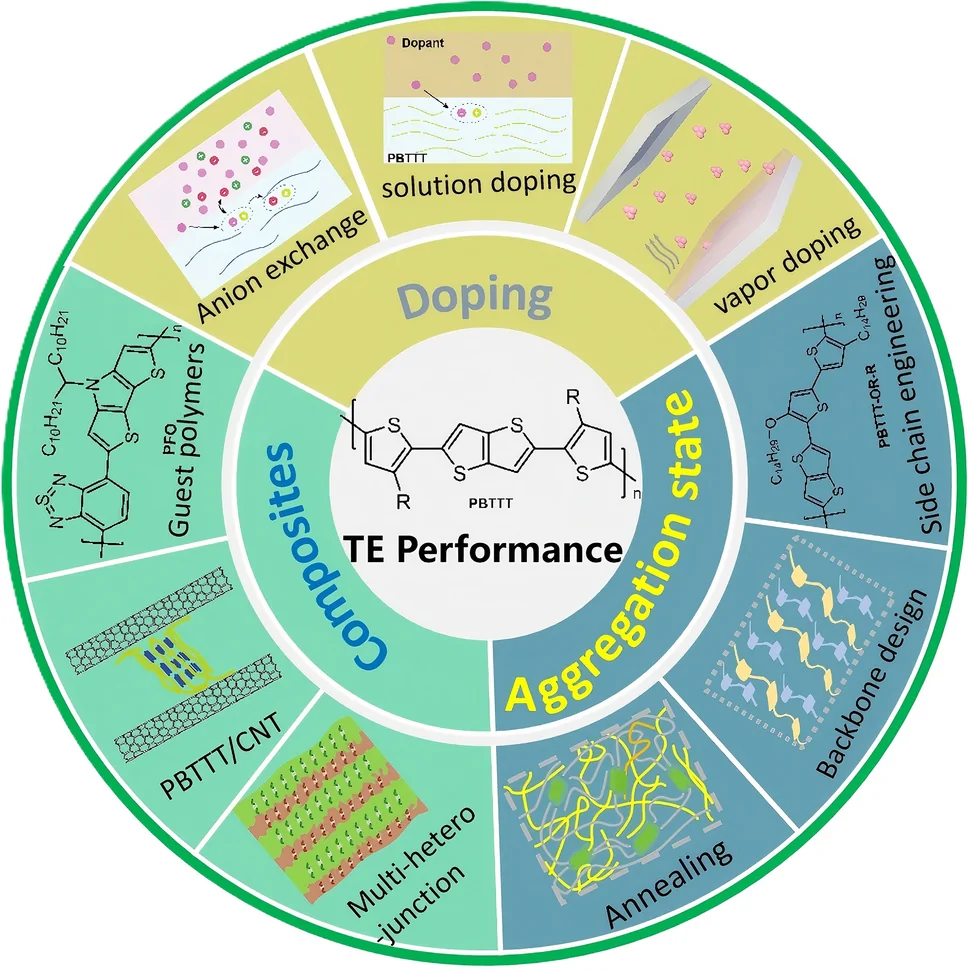
An overview of methods to enhance TE properties of PBTTT

In recent years, a class of conjugated polymers with liquid-crystalline phases, poly(2,5-bis(3-alkylthiophen-2-yl) thieno[3,2-b] thiophenes) (PBTTT) [48], has attracted increasing attention due to its unique molecular structure and excellent performance. As shown in Table 1, compared to these common TE polymers, PBTTT exhibits distinct advantages. Firstly, it possesses a rigid and highly conjugated backbone, which facilitates efficient charge transport and thereby significantly enhances the carrier mobility. Secondly, it has high-temperature stability and can be prepared using melt-processing technology. More importantly, PBTTT exhibits liquid-crystalline behavior through self-assembly of its extended thiophene backbones into highly ordered lamellar structures. This liquid-crystalline microstructure facilitates anisotropic charge transport properties critical for TE performance. The resulting long-range ordered domains emerge from solution processing, enabling efficient π–π stacking that enhances carrier mobility. Hence, PBTTT demonstrates versatile potentials in wide applications, including organic field-effect transistors [49, 50], organic photodetectors [51, 52], organic photovoltaics [53, 54], light-emitting diodes [55, 56], and TE devices [57, 58]. A recent study published by Jin et al. unveiled a PBTTT-based TE material that achieved a figure of merit of 1.28 at 368 K [59], clearly underscoring its promising potential for TE applications. So far, researchers have actively explored extensive strategies to enhance the TE performance of PBTTT. As illustrated in Fig. 1, these approaches encompass chemical doping to optimize charge carrier concentration and mobility, aggregation state regulation to fine-tune molecular ordering and crystallinity, and the development of composite materials that synergistically combine PBTTT with other functional components. Each of these methods has demonstrated unique potential in addressing the intrinsic limitations of PBTTT, paving the way for its promising prospects in next-generation TEs.

**Table 1:** Comparison of PBTTT with other polymer TE materials

	Structural characteristics	TE properties		Processability
		*σ*	*S*	
PEDOT:PSS	Rigid conjugation	High	Low	Solution
PTh	Linear conjugation	Medium	High	Vapor deposition
PANI	Weak conjugation	Low	Medium	Solution In-situ polymerization
PPy	Non-planar conjugation	Medium	Medium	Electrodeposition
PBTTT	Rigid conjugation Liquid-crystalline phases	High	Medium–High	Solution Melt

Despite significant advancements in PBTTT research for TE applications, a systematic review, covering the latest developments is strongly desired for this cutting-edge field. The most recent related review, published by our group in 2021 [60], can no longer reflect the current advances. Here, we aim to comprehensively summarize recent achievements in improving the TE performance of PBTTT, systematically compare the advantages and disadvantages of present approaches, and establish connections among various strategies to provide theoretical guidance and technical support for further optimization of PBTTT's TE performance. In the first section, we will systematically introduce the synthesis strategies of PBTTT and its derivatives, as well as film fabrication techniques, with particular emphasis on solution-processed approaches that represent the most prevalent form for practical applications. Then, we focus on discussing several representative approaches to enhancing TE performance. Subsequently, we provide a comprehensive discussion of the latest research advancements in PBTTT TE materials. Finally, based on the current research landscape, we offer an outlook on the future development trends of PBTTT and delve into the potential breakthrough directions for its TE properties. This review will offer researchers a comprehensive reference framework and promote the practical applications of PBTTT in TE materials.

## Fabrication

### Synthesis Method of PBTTT and Its Derivatives

McCulloch and colleagues proposed the synthesis of PBTTT based on a microwave-assisted Stille coupling reaction, being an important method to synthesize polythiophenes [61]. The Stille coupling reaction has become a widely used coupling reaction in organic synthesis. In the reaction, organotin compounds are coupled with halogenated alkanes or aromatic compounds under Pd catalysis [62, 63]. An exchange reaction between organotin compounds and halogenated species can facilitate the formation of new carbon–carbon bonds. The term “microwave-assisted” refers to the use of microwave radiation as an alternative to conventional heating methods. To date, microwave-assisted synthesis has been widely adopted in organic chemistry [61], primarily due to its significant advantages over traditional thermal approaches, including dramatically accelerated reaction rates along with higher product yields and reduced byproduct formation.

**Fig. 2:**
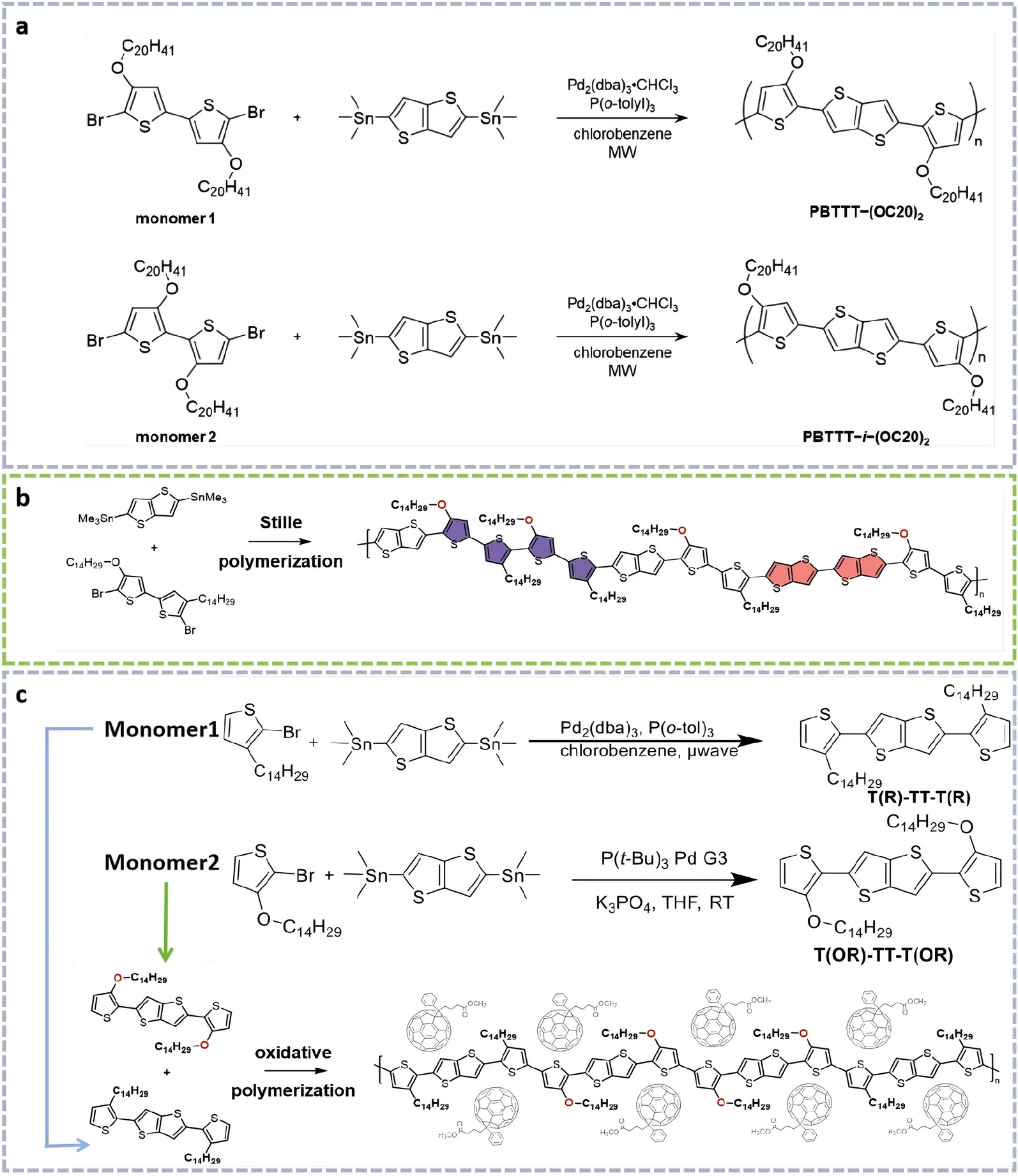
Synthesis methods of PBTTT derivatives. **a** Synthesis methods of PBTTT-(OC_20_)_2_ and PBTTT-i-(OC_20_)_2_ [65]. **b** PBTTT-OR-R synthesized by the Stille cross-coupling approach [68]. **c** PBTTT-OR-R synthesized by the symmetric oxidative polymerization approach [68]

As a highly designable conjugated polymer, PBTTT enables precise tailoring of material properties through rational side-chain engineering. Recent studies demonstrate that strategically modifying side-chain architecture allows effective controls intra/interchain interactions to enhance TE performance. Researchers have developed various PBTTT derivatives with functionalized side chains. Durand et al. [64] synthesized PBTTT-^*x*^O derivatives featuring single-ether-based side chains (*x* = 3, 5, 8, 11) and demonstrated that the single-ether moiety enhanced polymer/dopant interactions while simultaneously maintaining PBTTT's advantageous lamellar structure. Okamoto and coworkers [65] synthesized two PBTTT isomers with alkoxy (icosyl) side chains at distinct positions, PBTTT-(OC_20_)_2_ and PBTTT-i-(OC_20_)_2_ (Fig. 2a). These alkoxy substituents enhanced backbone rigidity through intramolecular non-covalent S···O interactions while elevating the highest-occupied-molecular-orbital (HOMO) energy levels compared to their alkyl counterparts. Subsequent study by Chen’s group [66] revealed that biaxial conjugated scaffolds with thienyl-ester functionalities could amplify charge transport properties under mechanical strain, achieving carrier mobility enhancements of up to threefold through ester-modified topological control.

However, the conventional Stille coupling approach for PBTTT derivatives often suffers from substantial homocoupling defects. To address this issue, Vanderspikken et al. [67] developed a symmetric oxidative polymerization method that effectively mitigated such defects. Subsequently, Goderis and coworkers [68] successfully extended this strategy to synthesize the PBTTT-OR-R derivative, previously regarded as highly desirable for its performance characteristics. For comparative evaluation, classical Stille cross-coupling was also employed to prepare the same polymer (Fig. 2b, c). Structural analyses demonstrated that the oxidative polymerization-derived PBTTT-OR-R exhibited superior chemical stability compared to its Stille-coupled counterpart.

### Fabrication Methods of PBTTT Films

**Fig. 3:**
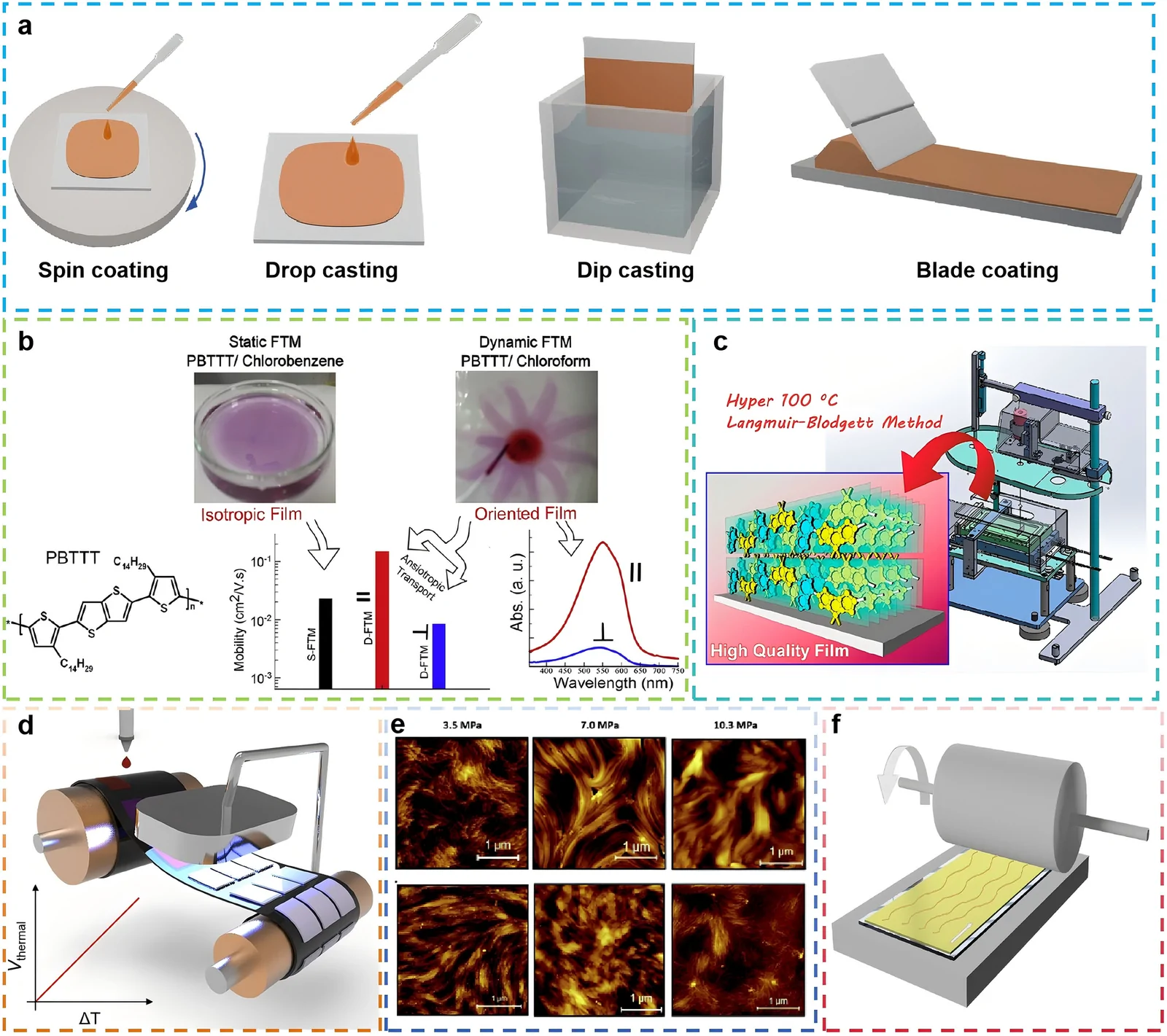
Processing methods for PBTTT films. **a** Traditional processing method, including spin coating, drop casting, dip casting and blade coating. **b** Schematic of floating film transfer method [76]. **c** Schematic of hyper 100 °C LB method [79]. **d** Schematic of nozzle printing [80]. **e** AFM images of PBTTT-C_14_ films formed in supercritical fluids under different pressure [81]. **f** Schematic of alignment by high-T rubbing [82]

Solution processing is the primary manufacturing approach for conjugated polymers. Typical solution processing techniques (Fig. 3a) for PBTTT encompass spin coating [69–71], drop casting [72], dip casting [73] and blade coating [74, 75]. Spin coating deposits a PBTTT solution onto a substrate, which is rapidly spun to form a film. However, spin-coated films typically exhibit disordered molecular packing, requiring post-deposition thermal annealing to improve crystallinity. Drop casting is simple, involving natural drying of a solution on a substrate. Nevertheless, it often produces films with poor uniformity and uncontrollable thickness. Dip coating involves immersing a substrate into a precursor solution and withdrawing it at a controlled rate. The resulting film morphology is highly dependent on withdrawal speed, solution concentration, viscosity, immersion time and withdrawal angle, affording reproducibility challenging. Blade coating applies shear forces to align molecules by spreading ink uniformly across a substrate using a bladed applicator, which often yields better molecular alignment than spin coating due to induced directional shear.

In addition to traditional fabrication methods, various innovative thin-film deposition techniques have been developed, displaying distinct characteristics and unique advantages.

#### Floating Film Transfer Method (FTM)

The FTM technique, proposed by Takashima’s research group [76–78] (Fig. 3b), represents a novel thin-film fabrication method based on liquid-interface self-assembly mechanisms. This approach ingeniously exploits the Marangoni effect, driven by surface tension gradients: when a PBTTT solution is drop-cast onto a liquid substrate, the rapid evaporation of solvent generates a concentration gradient, and induces radial fluid flow that facilitates uniform spreading. This method combines operational simplicity and low-cost fabrication, achieving both near-100% material utilization efficiency and the formation of high-quality and uniform thickness films.

#### Hyper 100 °C Langmuir–Blodgett (LB) Technique

Watanabe’s team [79] developed the Hyper 100 °C LB technique (Fig. 3c), successfully overcoming the limitations of conventional LB methods for amphiphilic molecules. By employing high-boiling-point solvents (e.g., ethylene glycol, boiling point 197 °C) or ionic liquids (boiling point > 300 °C) as the subphase, they achieved ordered monolayer transfer of PBTTT onto substrates via horizontal lifting at elevated temperatures (80–140 °C). This modified approach not only eliminates molecular misalignment caused by vertical lifting, but also significantly enhances film uniformity. More importantly, edge-on molecular packing mode is generated to facilitate efficient charge transport, a configuration where the π-conjugated backbone planes of the polymer chains are perpendicular to the substrate. In contrast, the face-on orientation features π-conjugated backbone planes parallel to the substrate.

#### Nozzle Printing Method

The nozzle printing fabrication technique developed by Boseok Kang’s team [80] achieved seamless integration of thin-film preparation and printing processes. They employed triphenylsulfonium triflate (TPS-TF) as a photoactivated dopant, which remains chemically inert in solution and only initiates solid-state doping reactions upon UV exposure. By leveraging this mechanism, the technique effectively circumvents issues inherent to conventional dopants, such as solution aggregation and printhead clogging, while enabling quantitative control of carrier concentration through precise UV exposure time modulation.

#### Physical Supercritical Fluid Deposition (p-SFD)

Kaake and coworkers [81] devised the p-SFD technique employing n-pentane (critical temperature: 196.6 °C, critical pressure: 3.37 MPa) as an inert supercritical medium to deposit polymer films through pressure-regulated phase transitions. By precisely controlling working pressure and solvent additive composition, this method enables the flexible fabrication of films with either nanowire structures or uniform smoothness. Although this green fabrication technique demonstrates promising application potential, its industrialization still faces key challenges, including equipment costs and process standardization.

#### High Temperature Rubbing (High-T Rubbing)

Based on the unique thermotropic liquid-crystalline behavior of PBTTT within the temperature range of 140–180 °C, the Brinkmann research group proposed an efficient high-T rubbing [82] alignment technique. Within this characteristic temperature window, PBTTT polymer chains exist in a dynamically ordered state, where the melting of side chains reduces intermolecular steric hindrance, enabling long-range alignment of backbone chains under shear forces. By precisely controlling rubbing temperature and shear rate, this method effectively overcomes van der Waals interactions, guiding PBTTT polymer chains to align along the rubbing direction, thereby forming well-ordered π-π stacking structures with mixed face-on/edge-on crystalline domains. This solvent-free solid-state alignment technique has demonstrated several advantages, including compatibility with roll-to-roll continuous manufacturing, shorter processing time compared to traditional epitaxial growth methods, and the ability to achieve uniaxial alignment films on conventional glass/plastic substrates without requiring single-crystal templates.

These fabrication strategies not only broaden the processing approaches for PBTTT films, but also offer versatile technological alternatives for performance enhancement and industrial manufacturing of organic electronic devices, marking a promising developmental trajectory in materials processing science.

## Strategies for Enhancement of TE Properties

The most important TE performance indicator is the figure of merit (*ZT*), which is expressed as:

1 $$ZT=\frac{{S}^{2}\sigma }{\kappa }T$$

where *S* denotes the Seebeck coefficient, *σ* is the electrical conductivity, and *S*^2^*σ* is called power factor (PF). *T* is the temperature, and *κ* is thermal conductivity. Over years of development, the *ZT* of PBTTT has significantly improved from an initial value of far below 0.1 to approximately 1.28 at 368 K [59]. This remarkable enhancement has been achieved through optimized strategies addressing three key parameters: *σ*, *S*, and *κ*. Below, we summarize the typical strategies for enhancement of TE properties.

### Doping

Doping represents a cornerstone strategy to optimize TE performance in conjugated polymers. Controlled dopant incorporation enables precise modulation of charge carrier density and crystallinity, directly optimizing key TE parameters, including *σ*, *S*, and *κ*.

#### Dopants for PBTTT

**Fig. 4:**
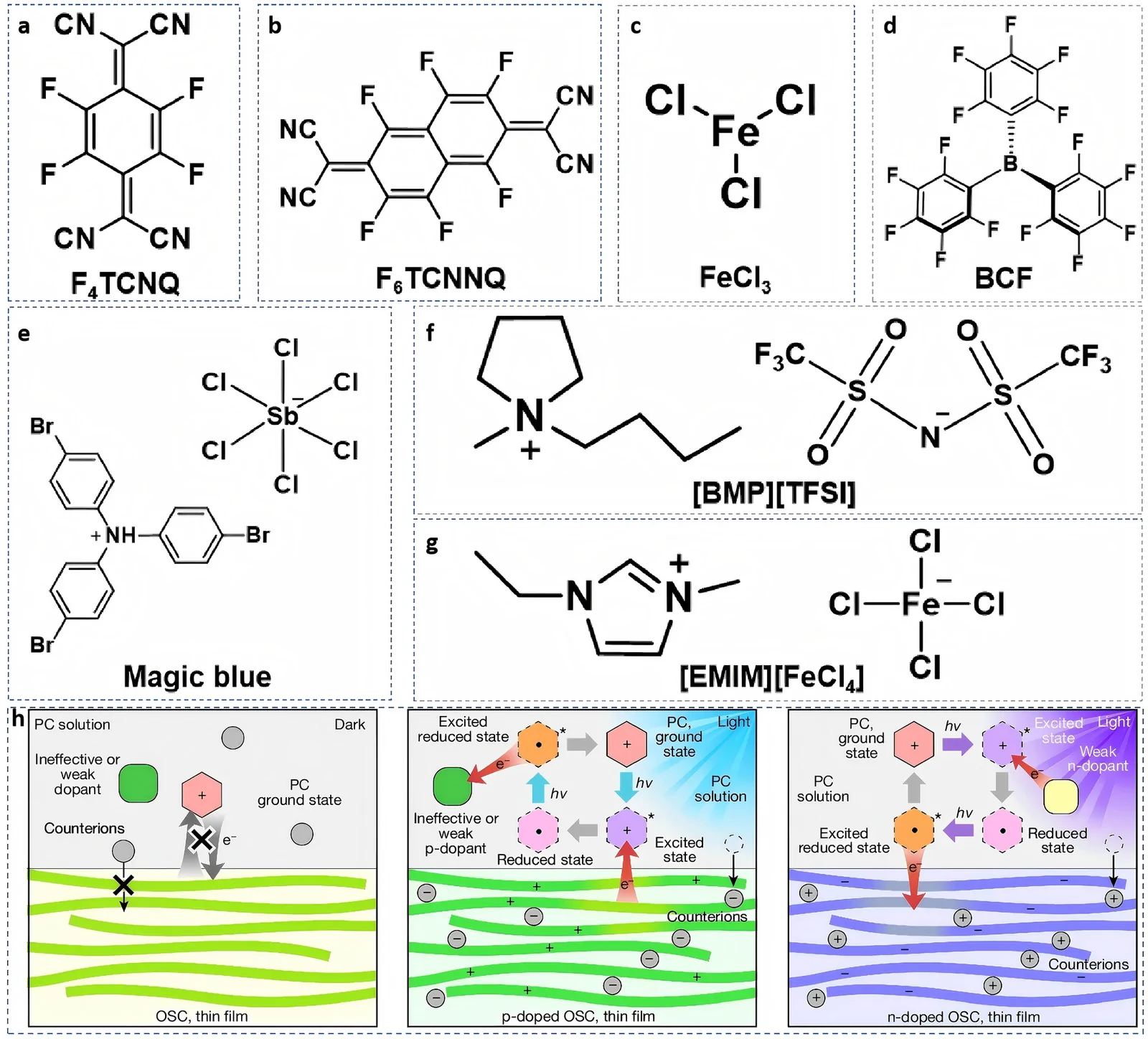
**a**–**g** Typical dopants for PBTTT, including F_4_TCNQ, F_6_TCNNQ, FeCl_3_, BCF, MB, and ILs. **h** Schematics of the photocatalytic doping processes [95]

In available literatures, PBTTT has been mainly studied in the context of p-type doping due to its favorable hole transport properties. Figure 4a–c displays different types of dopants typically employed in PBTTT. The doping mechanism fundamentally relies on redox-driven charge transfer, facilitated by favorable energy-level alignment between the dopant and the host material. As such, the p-doped PBTTT systems typically employ strong electron-accepting dopants whose energy levels complement those of PBTTT [83], such as 2,3,5,6-tetrafluorotetracyanoquinodimethane (F_4_TCNQ) [58, 84, 85], 1,3,4,5,7,8-hexafluoro-tetracyano-naphtho-quinodimethane (F_6_TCNNQ) [82], and ferric chloride FeCl_3_ [75].

Recent studies have expanded doping strategies to include acid–base chemistry as an alternative to traditional redox-based methods. Among these, the Lewis acid tris(pentafluorophenyl)borane (BCF) [73, 86, 87] has emerged as a particularly promising dopant (Fig. 4d). Its hydrated form, BCF-water, further acts as a strong Brønsted acid, enabling efficient proton-mediated doping. Compared to conventional electron acceptors like F_4_TCNQ, BCF offers superior solubility in various organic solvents, facilitating homogeneous doping and improved charge transfer efficiency. Recent work has successfully demonstrated BCF as an effective p-type dopant for PBTTT, achieving high *σ* and excellent charge transport properties.

Since high doping concentrations can degrade the material's microstructure and stability by disrupting the π-π stacking of polymer chains, thereby negatively impacting conductivity, Brinkmann et al. employed tris(4-bromophenyl)ammoniumyl hexachloroantimonate as a dopant, commonly known as Magic Blue (MB) [88–90] (Fig. 4e). MB preferentially localizes in the amorphous regions of PBTTT, while charge carriers (holes) predominantly concentrate within the crystalline domains. Crucially, the crystalline phase remains undoped, preserving its high charge carrier mobility. In contrast, conventional dopants such as F_4_TCNQ and F_6_TCNNQ intercalate into the side-chain layers of the crystalline phase, disrupting the critical π–π stacking and consequently reducing conductivity. Remarkably, this strategy achieved an exceptional conductivity of 9700 S cm^−1^ with MB doping [88], significantly outperforming F_6_TCNNQ-doped samples which showed only 2430 S cm^−1^.

In addition, ionic liquids (ILs) [91–94] have proven to be highly effective dopants for conjugated polymers due to their exceptional properties, including superior environmental stability, broad solvent compatibility, and the ability to facilitate ion–electron coupled transport while minimizing structural disruption to crystalline domains. ILs consist of cation–anion pairs, where the anion acts as an oxidizing agent, enabling p-doping via charge transfer, while the cation provides an ion-conductive medium, enhancing ion diffusion. Tanaka et al. [92] developed an IL-based chemical doping strategy using [1-ethyl-3-methylimidazolium (EMIM)][FeCl_4_] (Fig. 4e). X-ray diffraction (XRD) confirmed the retention of high crystallinity after IL doping, avoiding the structural degradation typically induced by conventional dopants. Sirringhaus and colleagues [93] employed a synergistic doping strategy through the combination of [1-butyl-1-methylpyrrolidinium] [bis(trifluoromethanesulfonyl)imide] ([BMP][TFSI]) (Fig. 4g) with FeCl_3_, which not only enhanced doping efficiency but also maintained PBTTT crystallinity while exhibiting minimal structural degradation.

A recent study published in Nature [95] introduces a photocatalytic doping approach for organic semiconductors, where oxygen serves as a mild p-type dopant. In Fig. 4h, the method employs photocatalysts, which, upon light excitation, extract electrons from conductive polymers, undergo self-reduction, and are subsequently regenerated by O_2_. Unlike conventional doping methods that often disrupt crystallinity, this strategy maintains thin-film molecular ordering and crystallinity during the doping process. Their approach can operate under mild and environmentally benign conditions, enabling reactions to proceed at room temperature in ambient air. Remarkably, only trace amounts of salts are consumed to maintain charge neutrality, while the photocatalysts remain fully recyclable, minimizing chemical waste and enhancing process sustainability.

#### Doping Methods

Doping methods can be systematically classified based on their implementation strategies, with the most extensively studied approaches encompassing: Solution doping [58, 92, 96, 97] and vapor doping [72, 84]. Most recently, anion exchange doping [85, 93, 94] has been recognized as an advancement in doping technology, offering unique advantages in terms of tunable charge carrier density and material stability. Solution doping is a processing technique that introduces dopant into a host material by co-dissolving both components in a common solvent. This method features straightforward operation and demonstrates excellent compatibility with broader manufacturing processes. Vapor doping is a dry-phase processing technique where gaseous dopant diffuse into solid-state materials, enabling precise control over dopant concentration and penetration depth. Anion exchange is an electrochemical process where mobile ions in a solution are reversibly substituted with counterions bound to a solid material (ion exchanger). This occurs via electrostatic interactions while preserving charge neutrality.

##### Solution Doping

Solution doping remains the most prevalent doping approach due to its operational simplicity. However, achieving effective carrier generation typically necessitates high dopant loadings that compromise the crystalline integrity of PBTTT, a trade-off that fundamentally limits TE performance. Solution-sequential (SSq) doping was designed specifically to decouple dopant diffusion from crystallization kinetics [58, 92, 97], wherein dopants are introduced to pre-deposited PBTTT films rather than blended directly in solution. Kilwon Cho et al. [58] employed SSq doping to incorporate F_4_TCNQ into PBTTT, achieving a maximum dopant concentration of 20 mg mL^−1^ with a resulting conductivity of 13 S cm^−1^. However, a key limitation persists: The restricted infiltration of F_4_TCNQ into the crystalline domains of PBTTT constrains doping efficiency and ultimately caps conductivity enhancement.

**Fig. 5:**
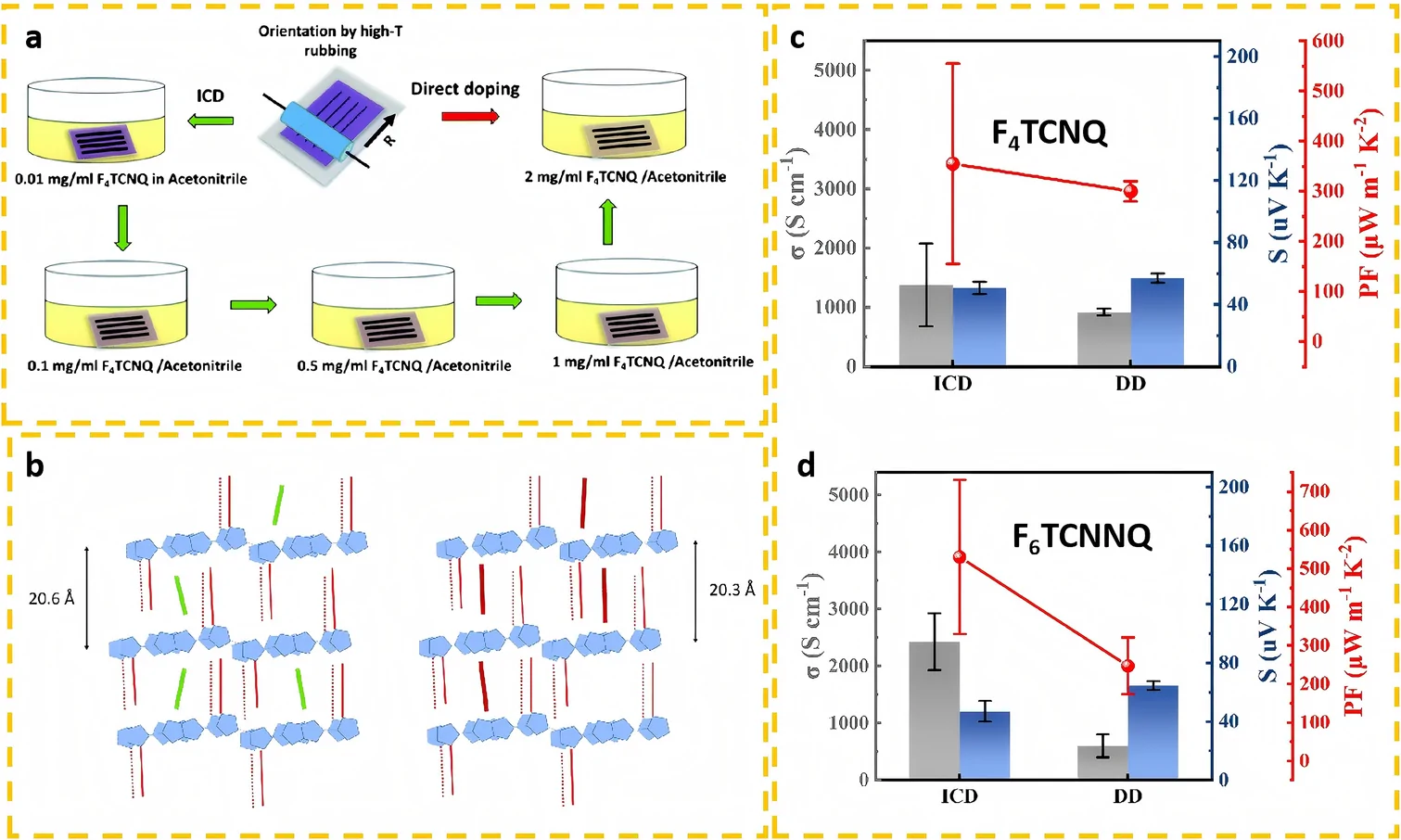
Experimental process and data of the ICD method [96]. **a** Schematic of ICD method. **b** Schematic of the dopant intercalation of F_4_TCNQ and F_6_TCNNQ in the layers of alkyl side chains. **c** Comparison with ICD and DD doped with F_4_TCNQ. **d** Comparison with ICD and DD doped with F_6_TCNNQ

Subsequently, Brinkmann and coworkers [96] introduced an incremental concentration doping (ICD) strategy, systematically comparing the performance of two common p-type dopants, F_4_TCNQ and F_6_TCNNQ. As illustrated in Fig. 5a, the ICD method employs sequential, stepwise immersion into solutions with progressively increasing dopant concentrations, whereas direct doping (DD) utilizes single-step immersion; this gradual ICD approach minimizes disruptions to PBTTT’s molecular order, preserving high charge carrier mobility while enabling deeper and more uniform dopant penetration into crystalline domains. However, this method yields limited TE enhancement for dopants with excessive diffusion rates. As shown in Fig. 5b, F_4_TCNQ demonstrated high diffusivity, being 4.5 times greater than that of F_6_TCNNQ, resulting in disordered dopant arrangements under ICD treatment; conversely, the larger molecular dimensions of F_6_TCNNQ facilitated ordered alignment. Consequently, ICD substantially enhanced TE performance for F_6_TCNNQ compared to DD. Figure 5c, d directly quantifies these performance gains: ICD achieved a 114% PF improvement for F_6_TCNNQ versus a marginal 18% increase for F_4_TCNQ.

##### Vapor Doping

**Fig. 6:**
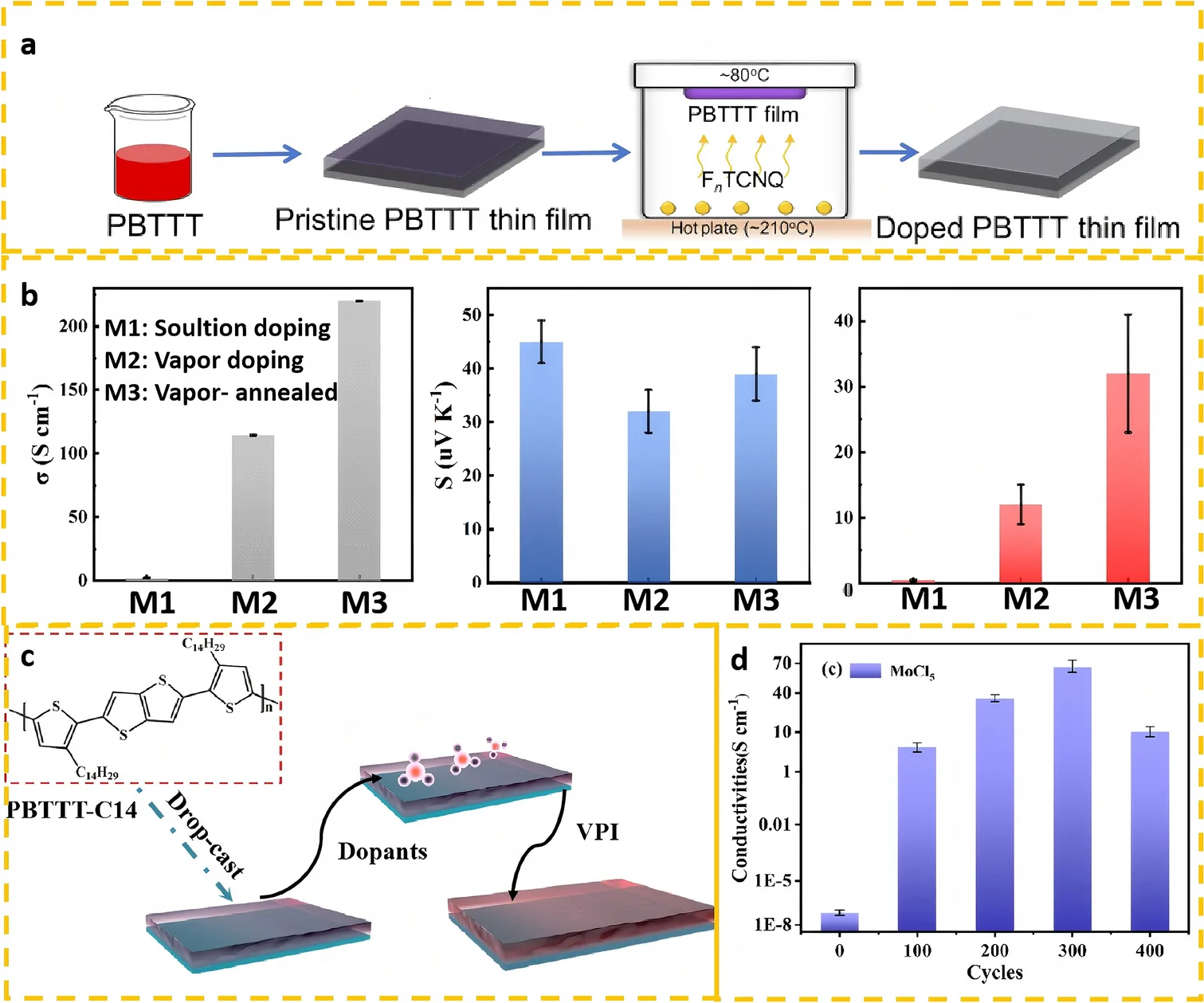
Schematic illustration of two typical vapor doping methods. **a** Schematic illustration of vapor doping process [84]. **b** A comparison of the effects of three treatment methods on TE performance, where M1 means solution doping, M2 denotes vapor doping and M3 refers to combination of vapor doping and annealed treatment [84]. **c** Schematic illustration of VIP. [72] and **d** *σ* of the PBTTT doped with MoCl_5_ by VIP method [72]

Vapor doping offers a solvent-free alternative to conventional solution-based processing. This technique introduces dopants in the vapor phase, minimizing structural and morphological degradation. Figure 6a schematically illustrates the vapor doping process, which avoids pre-aggregation issues inherent in solution environments, thereby reducing phase separation and structural defects. As shown in Fig. 6b, Chabinyc et al. [84] compared the TE performance of solution-doped and vapor-doped films, finding that vapor doping achieved a over 50-fold enhancement in the *σ* of PBTTT over solution doping. Furthermore, annealing further significantly increased conductivity. Analysis of orientational correlation lengths demonstrated that vapor-doped films exhibited enhanced molecular alignment of the PBTTT polymer chains. Complementary work by Cho’s group [58] revealed that vapor doping enables simultaneous dopant incorporation in both amorphous and crystalline domains, while solution doping predominantly deposits dopants in amorphous regions.

For inorganic dopants, the vapor-phase infiltration (VPI) technique has been developed as an effective doping approach [72, 98] (Fig. 6c). Derived from atomic layer deposition, VPI enables self-limiting reactions between metallic precursors and oxidants [99, 100], thereby strengthening the bonding between inorganic dopants and organic matrices. Wang et al. [72] synthesized hybrid PBTTT-C_14_ films via controlled in situ VPI using MoCl_5_ and TiCl_4_ as precursors. Owing to the strong Lewis acidity of MoCl_5_, which efficiently extracts electrons from the PBTTT backbone, the MoCl_5_-doped PBTTT films achieved a maximum conductivity of 67.1 S cm^−1^ (Fig. 6d). Compared to conventional vapor doping methods, VPI method induces less perturbation to the chain conformation of polymer molecules. The orientational correlation length remains closer to its intrinsic state, and the doping depth offers greatly enhanced controllability.

##### Anion Exchange Doping

**Fig. 7:**
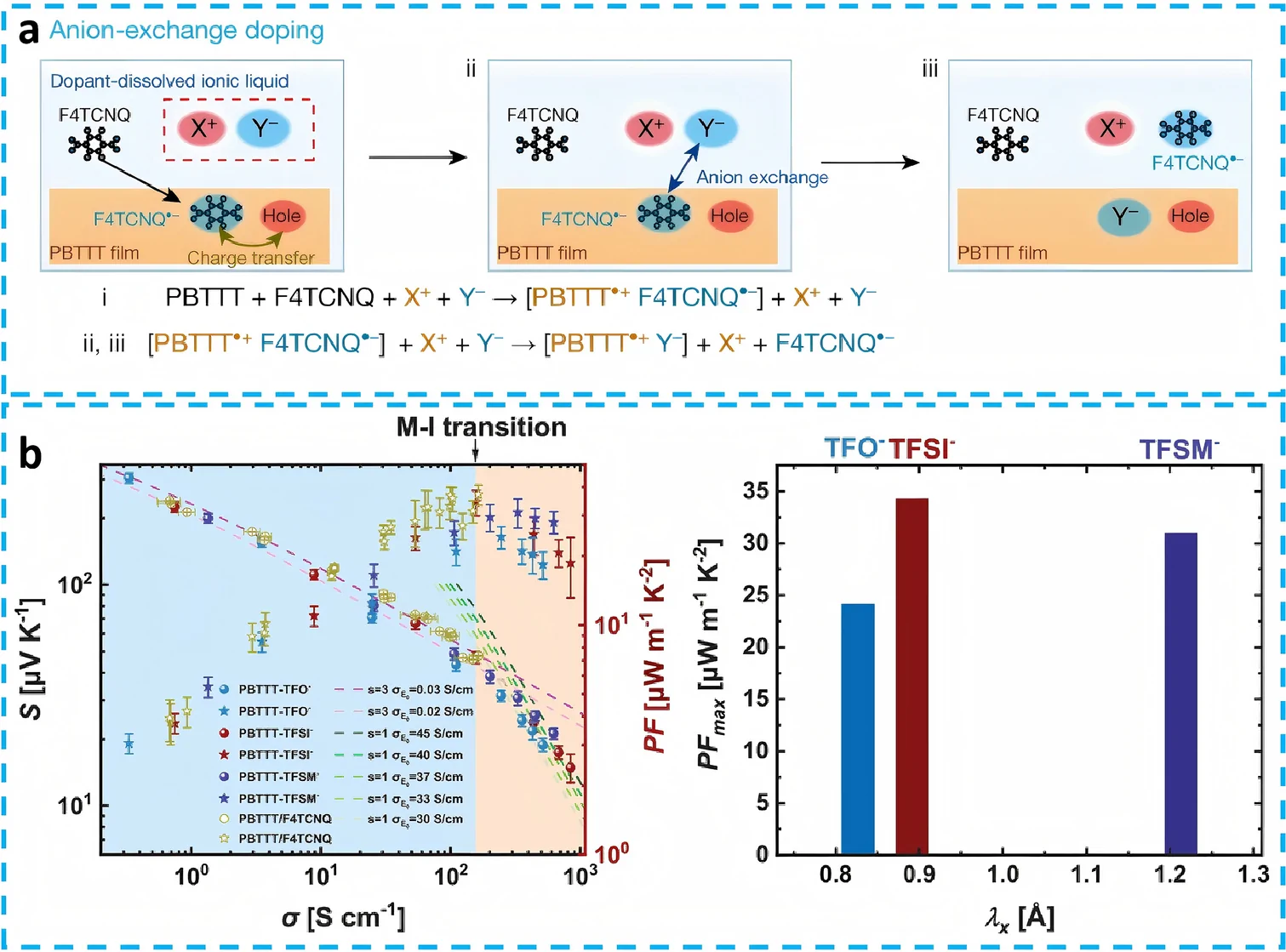
Schematic illustration anion exchange doping. **a** Schematic illustration of anion exchange doping [85]. **b** In anion exchange doping, the effect of ion size on TE properties, using three different types of ions, i.e., TFO^−^, TFSI^−^, TFSM^−^ [94]

Doping efficiency and charge carrier concentration are principally governed by the electrochemical redox potential difference between the π-conjugated polymer host and the dopant species [101]. For high-efficiency p-type doping, the electron affinity (EA) of the dopant must at least match the ionization potential (IP) of the polymer [102]. However, increasing the EA of dopants often compromises chemical stability, severely restricting viable dopant selection. To circumvent this limitation, Watanabe et al. [85] proposed an anion exchange doping approach (Fig. 7a), utilizing F_4_TCNQ as the p-type dopant and [EMIM][TFSI] as the charge transfer mediator. Figure 7a schematically illustrates the procedural workflow of anion exchange doping, which has two key features. First, the process is mediated by an IL solvent, where conventional small p-type dopant anions effectively and instantaneously exchange with a second anion provided by the IL. Introducing optimized ion salts into conventional binary donor–acceptor systems can overcome the oxidation–reduction potential limitations described by the Marcus theory, allowing for an anion exchange efficiency close to 100% and a *σ* of up to 620 S cm^−1^. Secondly, by adjusting the physicochemical properties of the additional anion, the thermal durability of the film can be further enhanced.

A mechanistic investigation of anion exchange doping was conducted by Sirringhaus et al. [93] to elucidate the fundamental processes governing this phenomenon. FeCl_3_ was employed as the chemical dopant in conjunction with [BMP][TFSI] as a representative ion exchange electrolyte, enabling comprehensive analysis of the doping equilibrium and kinetic parameters in PBTTT films. Notably, the selection of acetonitrile as the processing solvent was found to substantially mitigate electrolyte association effects while simultaneously optimizing doping efficiency. By optimizing solvent selection and enhancing oxidizer strength, the thermodynamic constraints of traditional doping are overcome, leading to a further increase in the doping efficiency, hence, the *σ* of PBTTT can reach 1120 S cm^−1^.

Subsequently, Sirringhaus et al. investigated the influence of counterion size on TE performance [94]. They employed three anions of differing sizes: the bulky tris(trifluoromethylsulfonyl)methide anion (TFSM^−^), the moderately sized bis(trifluoromethylsulfonyl)imide anion (TFSI^−^), and the relatively small trifluoromethanesulfonate anion (TFO^−^). The TE performance results are presented in Fig. 7b. Crucially, these results revealed only a weak dependence of the TE performance on anion size. Instead, the study identified that optimizing the spatial distribution of dopant ions within the film was key to achieving higher *ZT*.

### PBTTT Composites

In addition to doping, fabrication of composites is another means of enhancing TE performance. In composite materials, two or more different types of materials are combined to leverage the advantages of each material and compensate for their respective shortcomings, achieving a synergistic effect where 1 + 1 > 2 [103, 104].

**Fig. 8:**
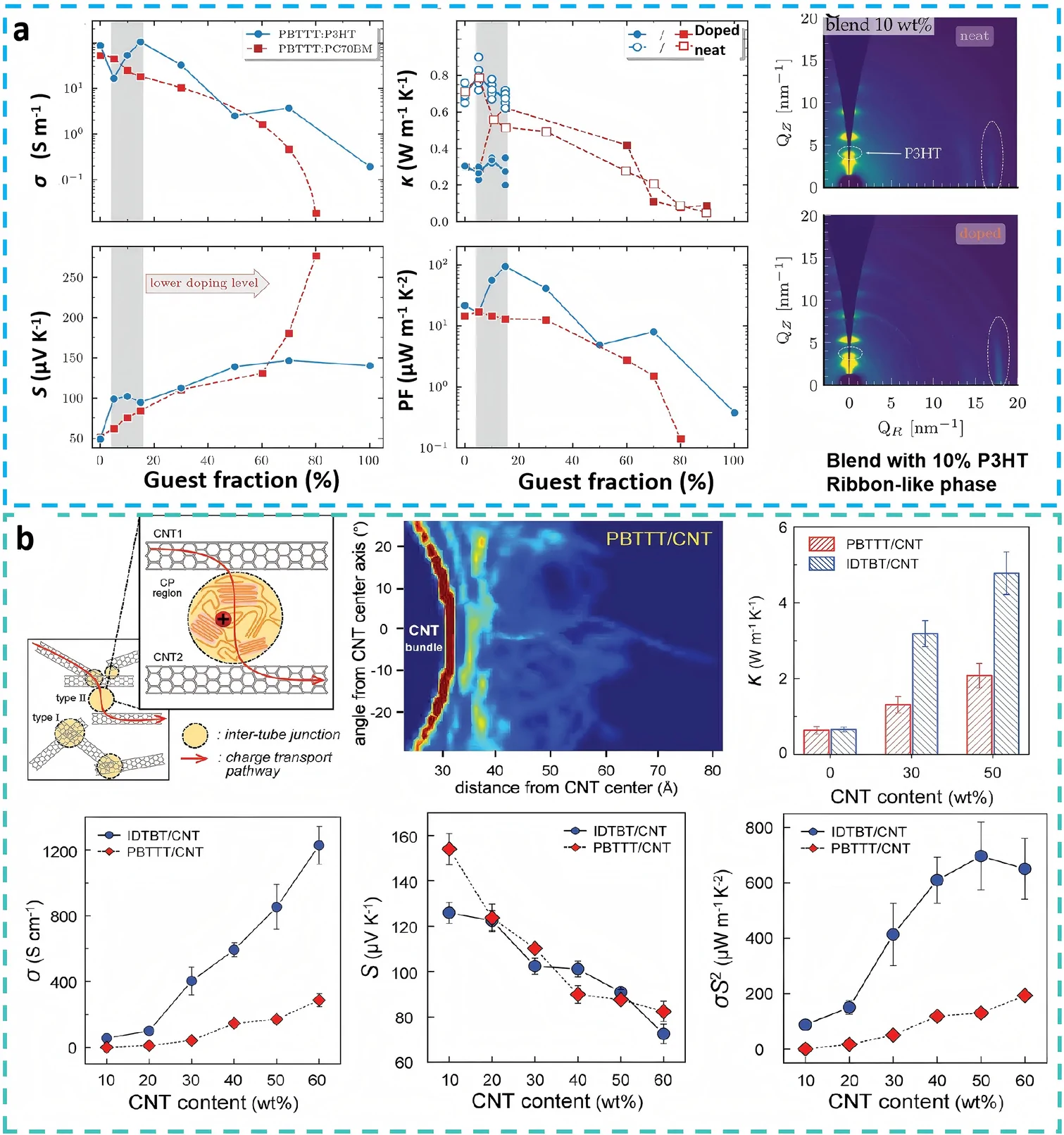
Study on PBTTT-based composites. **a** TE properties for films of PBTTT with varied fractions of P3HT and PC70BM, and GIWAX images for the PBTTT blended with 10% P3HT annealed at 180 °C [74]. **b** Comparison of the TE properties between PBTTT/CNT composites and IDTBT/CNT composites [106]

Campoy-Quiles et al. [74] incorporated guest polymers into a PBTTT matrix with a mass fraction of 10%–15%, systematically investigating their influence on the TE properties of the host polymer. The researchers systematically screened nine guest materials (including P3HT, fullerene derivatives, and multiple donor polymers), establishing a definitive composition-morphology-property relationship. As illustrated in Fig. 8a, incorporating 10 wt%–15 wt% P3HT into the host material PBTTT yields a remarkable five-fold enhancement of the PF compared to pristine PBTTT, achieving a *ZT* value of ~ 0.1. Further structural characterization revealed that annealing the 10 wt% P3HT blend at 180 °C spontaneously induces the formation of oriented ribbon-like nanostructures, analogous to the highly ordered phase observed in pure PBTTT annealed at a significantly higher temperature of 270 °C.

Carbon-based materials are dominant matrix candidates for flexible TE composites owing to their exceptional *σ* and mechanical robustness. Researchers have recently strategically incorporated PBTTT with carbon architectures, including carbon nanotubes (CNTs) [105, 106] and graphene [107]. However, limitations still persist in the PBTTT/CNT composite system. Cho et al. conducted a comparative investigation of PBTTT/CNT and indacenodithiophene-co-benzothiadiazole (IDTBT)/CNT composites using a combined experimental and molecular dynamics simulation, to elucidate interfacial charge transport mechanisms [106]. Analysis demonstrates that PBTTT tends to form thick aggregated shells on CNT surfaces (Fig. 8b), resulting in discontinuous charge transport pathways that hinder carrier hopping between adjacent CNTs, limiting *σ* to 169.9 S cm^−1^. In contrast, the IDTBT/CNT system achieves significantly higher *σ* (853.5 S cm^−1^) owing to continuous charge transport pathways.

To enhance the TE properties of PBTTT/CNT composites, structural modifications inspired by IDTBT can be implemented through backbone engineering, by introducing flexible copolymer units such as siloxane segments to reduce interchain stacking rigidity and designing branched side-chain architectures to improve molecular orientation freedom. In addition, surface modification for CNTs may achieve P3HT/PBTTT-like synergistic effects, strengthening π-π interactions with CNTs and facilitating interfacial charge tunneling.

### Aggregation State Controlling

The TE performance of PBTTT-based materials can also be significantly enhanced through the modulation of PBTTT chains’ aggregation states, including molecular packing and crystallization dynamics. A critical advantage lies in PBTTT's liquid-crystalline phase, which inherently facilitates structural alignment and enables more refined control over its aggregation behavior during processing.

Well-ordered molecular arrangements optimize charge transport properties, leading to higher *σ* and *S* by reducing carrier scattering and enhancing delocalization within π-conjugated systems [82, 96]. Simultaneously, fine-tuning the crystalline morphology, such as lamellar stacking, grain boundary density, and degree of crystallinity, effectively suppresses phonon transport through selective scattering mechanisms, thereby minimizing *κ* while preserving high electronic mobility [108, 109]. The key tunable variables governing aggregation state include: solvent engineering, molecular structure design and thermal treatment [110].

#### Solvent Engineering

Solution processability stands as a paramount advantage of conjugated polymers, enabling scalable fabrication of organic electronic devices through low-cost techniques [111–118]. Crucially, the solvent selection governs the crystallization kinetics and thermodynamic pathways during film formation, directly determining the TE performance of the resulting films. As evidenced by recent studies [112–114], the properties of solvents, boiling point, polarity, and solvent–solute interaction parameters, exert profound control over the crystallization behavior of PBTTT.

**Fig. 9:**
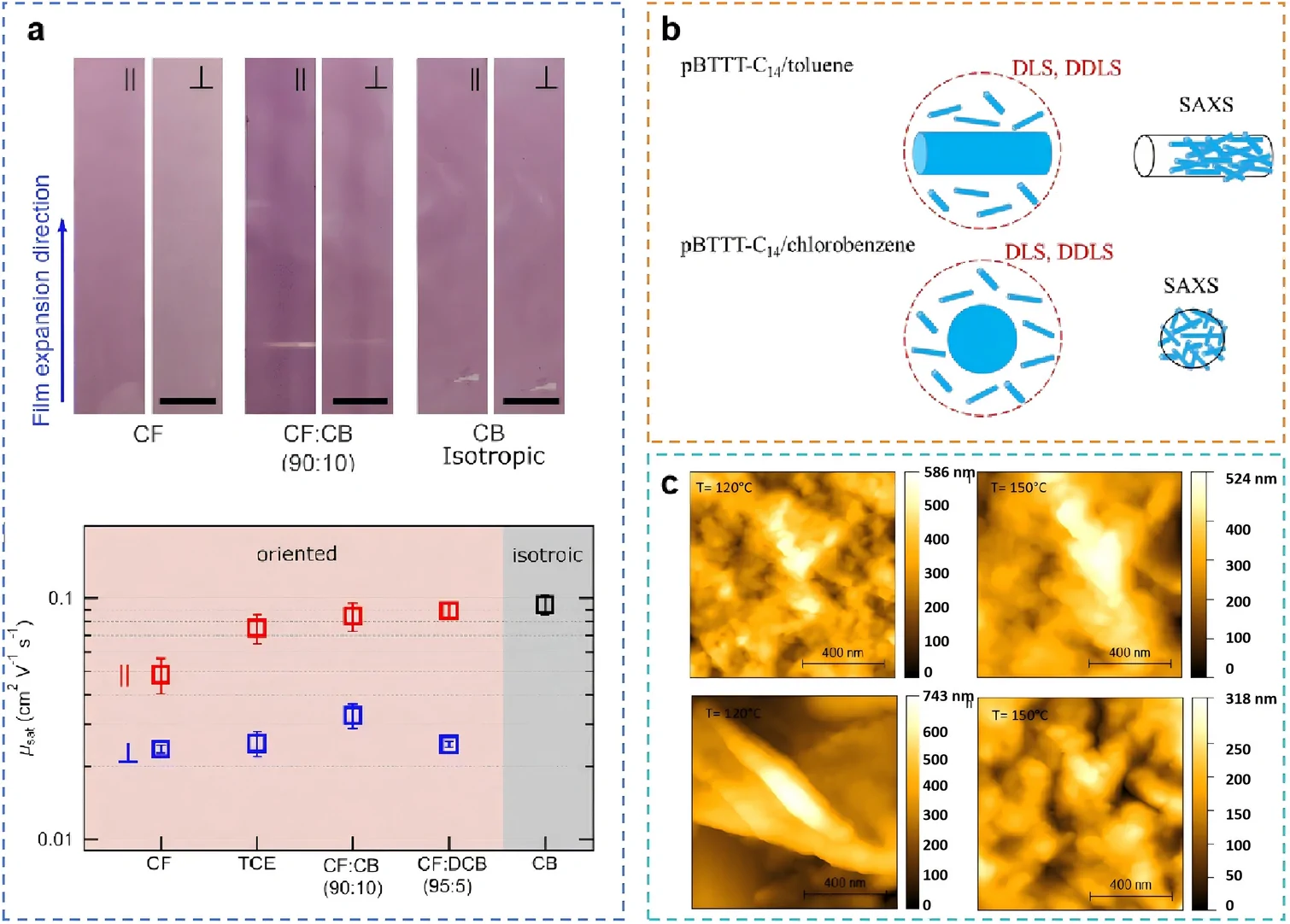
Effect of solvents on the crystallization behavior of PBTTT. **a** Polarized photograph of the large-area films processed with different solvents and the corresponding carrier mobility [77]. **b** Schematic illustrations of a hierarchy of structural features of PBTTT-C_14_ clusters incubated in two slightly different aromatic solvents [115]. **c** Solvent-dependent crystallization behaviors under 120 and 150 °C annealing temperature [117]

The study demonstrates that the boiling point of solvents plays a pivotal role in modulating both the morphology and film-forming mechanisms of PBTTT films via the FTM method [76]. For high-boiling-point solvents, the polymer solution spreads slowly across the aqueous substrate. During this static casting process, prolonged solvent evaporation allows thermodynamic self-assembly, resulting in isotropic films with edge-on molecular orientation. In contrast, low-boiling-point solvents initiate a dynamic casting regime: rapid solvent spreading, coupled with instantaneous evaporation, induces a transient lyotropic liquid-crystalline phase prior to solidification. In subsequent work [77], the researchers further elucidated how solvent boiling temperature dictates PBTTT’s crystallization behavior and charge carrier mobility. By comparing four solvents, i.e., chloroform (CF), trichloroethylene (TCE), chlorobenzene (CB), and 1,2-dichlorobenzene (DCB), they identified two distinct crystallization regimes: For low-boiling-point solvents (CF and TCE), rapid evaporation promotes kinetic trapping of PBTTT chains, yielding films with pronounced edge-on orientation; While for high-boiling-point solvents (CB and DCB), slow evaporation allows thermodynamic relaxation, leading to isotropic films. However, the correlation between mobility and crystallographic orientation follows a non-trivial trend (Fig. 9a). While low-boiling-point solvents yield better-aligned molecular packing, the resulting lower interchain coupling density leads to lower mobility. Conversely, high-boiling-point solvents produce isotropic films with reduced orientation but significantly enhanced π-π stacking density through face-on dominated morphologies, ultimately delivering superior mobility.

In addition to the influence of solvent boiling points on the aggregation morphology of molecular chains, solvent polarity has been demonstrated to exert a marked impact on these structural features. As evidenced by the work of Yi et al. [115], systematic investigations into PBTTT aggregation states in toluene (Tol) and CB revealed contrasting mesoscale architectures governed by solvent-mediated thermodynamic and kinetic controls. Their analyses revealed a obvious dichotomy in aggregation behavior (Fig. 9b): In Tol, the low-polarity solvent facilitates rapid torsional relaxation of polymer backbones, enabling efficient π-π stacking that drives the formation of anisotropic nanofibrils with aspect ratios > 3. In contrast, CB's higher polarity and steric hindrance from chlorine substituents disrupt ordered assembly, resulting in isotropic spherical aggregates with fractal dimensions < 2. This fundamental understanding of solvent-polymer dynamics provides critical guidelines for morphology control in solution-processed organic electronics. Gu and coworkers [118] further investigated the influence of solvent quality on PBTTT's aggregation behavior by analyzing CB and Tol systems through static light scattering experiments. By constructing Debye plots, they determined the second virial coefficients (A_2_), revealing that CB's positive A_2_ value classifies it as a good solvent that promotes polymer chain dissolution and dispersion, while Tol’s negative A_2_ value indicates poorer solvent quality that drives polymer chain aggregation. This comparative analysis demonstrates how solvent-mediated interactions critically regulate PBTTT’s molecular assembly, providing an insight for controlling film morphology in organic electronic applications.

Moreover, solvent selection critically governs the thermodynamics of the annealing process, particularly in modulating nucleation kinetics and long-range ordering. Abdoul et al. [117] explored the synergistic effects of solvent selection (Tol, CB, and tetrahydrofuran (THF)) and thermal annealing on the crystalline morphology of PBTTT films. Their study revealed distinct solvent-dependent crystallization behaviors: when processed with CB, PBTTT formed a continuous and uniform nanofibrillar network upon annealing, facilitating efficient charge transport. In contrast, both Tol and THF induced the formation of a terraced liquid-crystalline phase above 120 ℃ annealing temperatures, as shown in Fig. 9c.

#### Molecular Structure Design

##### Backbone Regulation

The regulation of polymer backbone architecture has been predominantly directed toward molecular weight (MW) optimization to govern charge transport mechanisms [69, 119–122]. Higher-MW polymers inherently promote extended chain conformations, fostering enhanced interchain alignment and long-range crystalline order while intensifying π-orbital overlaps. Fan et al. [69] established a rigorous correlation between MW escalation in PBTTT-C_14_ (MW: 11,867–175,199 g mol^−1^) and hierarchical structural ordering, observing a concurrent increase in π-π stacking and *σ* with rising MW, suggesting strengthened intermolecular interactions.

**Fig. 10:**
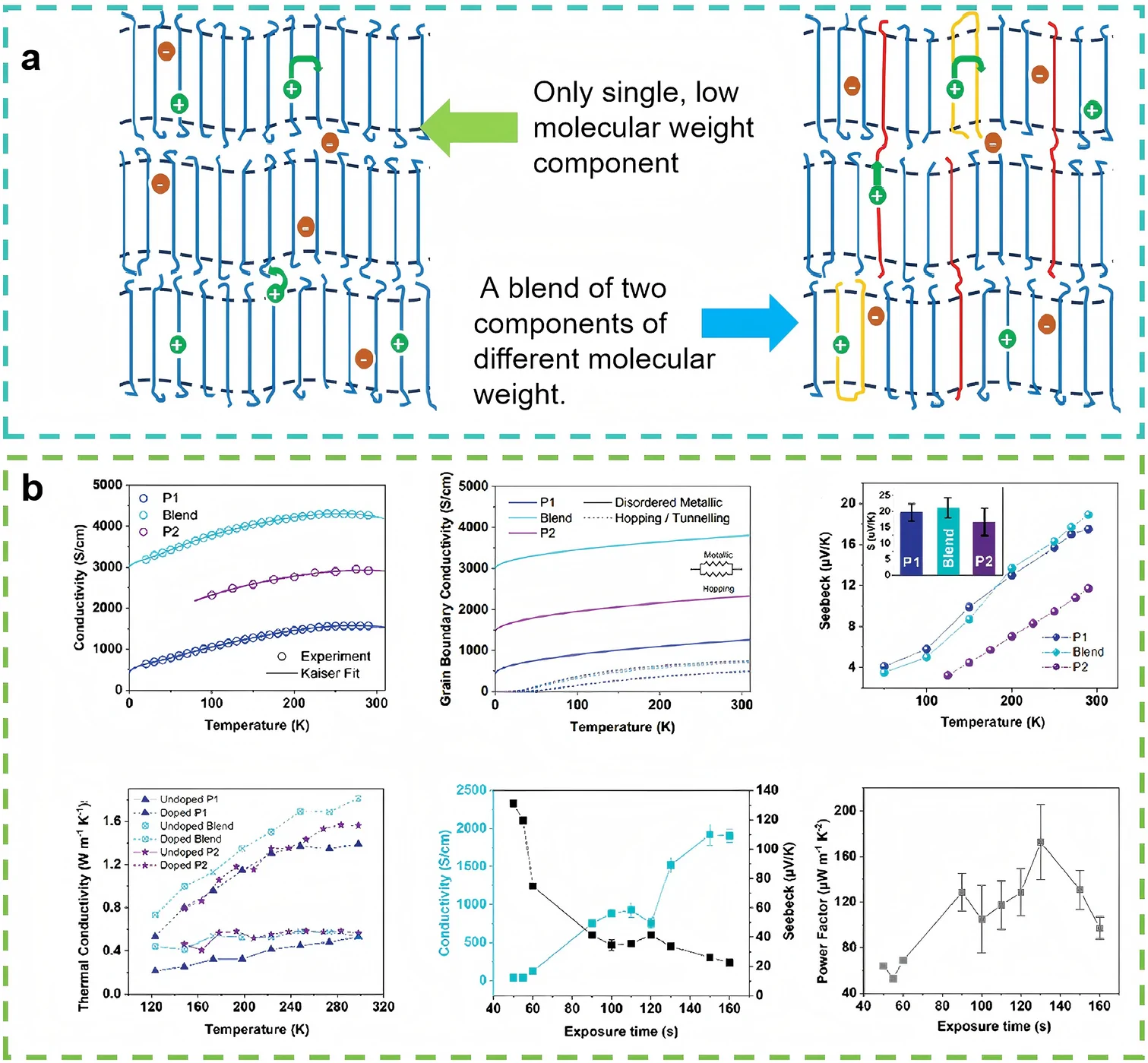
Illustration of the influence of polymer backbone on crystallization. **a** Schematic of the aligned ribbon-phase with a single, low molecular weight component (left) and with a blend of two components of different molecular weight (right). **b** TE related properties of the blended-PBTTT, including conductivity, *S* and *κ* [122]

Advancing beyond uni-MW paradigms, Zhu et al. [122] developed a bimodal MW distribution strategy employing controlled blends of high-MW and low-MW PBTTT fractions (Fig. 10a). The low-MW polymer functioned as tie-chains, bridging crystalline domains and improving inter-grain charge transport while preserving TE performance. This approach yielded a high *σ* of 4,810 S cm^−1^ and a PF of 173 μW m^−1^ K^−2^ (Fig. 10b), without compromising the *S* or excessively increasing *κ*.

The blending of PBTTT chains with different MWs aligns conceptually with the host–guest strategy proposed by Campoy-Quiles et al. [74], aiming to establish a continuous network of interconnected polymer chains. While current studies utilize binary blending, future work could explore ternary blends (e.g., low/medium/high MW) to construct a more continuous tie-chain network, potentially breaking through existing electrical conductivity bottlenecks.

##### Side-Chain Engineering

**Fig. 11:**
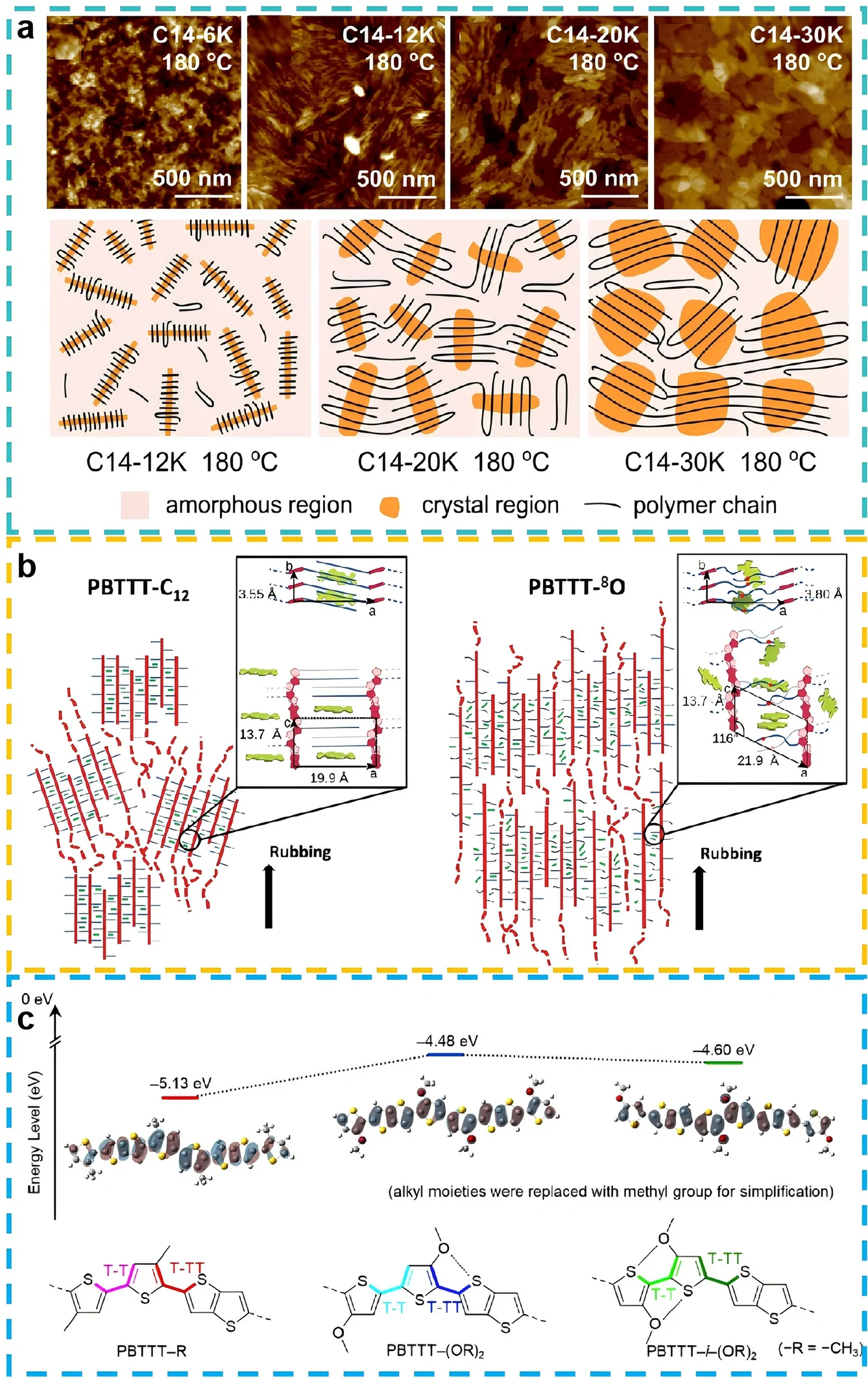
Illustration of the side-chain engineering. **a** AFM imaging of the modulation mechanism of MW and annealing temperature on the morphology and microstructure of PBTTT-C_14_ [126]. **b** Schematic illustration of the mesoscale order induced by rubbing in PBTTT-C_12_ (left) and PBTTT-^8^O (right) [127]. **c** Chemical structures and HOMO of PBTTT with alkyl side chains and alkoxyl side chains [65]

Beyond backbone modifications, side-chain engineering has emerged as a powerful approach to tailor polymer crystallization and electronic properties [65, 123–128]. Peng and coworkers [126] conducted systematic investigations of PBTTT derivatives functionalized with linear alkyl side chains ranging from hexyl (C_6_) to hexadecyl (C_16_). Through comprehensive characterization of crystalline structures and charge transport properties, they demonstrated that the combination of C_14_ side chains and high molecular weight synergistically promotes the formation of a highly ordered edge-on crystalline structure upon annealing at 180 °C (Fig. 11a), which exhibits optimal molecular alignment and achieves a remarkable hole mobility of 0.54 cm^2^ V^−1^ s^−1^.

Additionally, polar side chains can induce edge-on orientation of the polymer backbone. Polar ether-functionalized side chains (n-C_7_OC_4_) were incorporated in PBTTT (PBTTT-^8^O) by Durand et al. [127]. This modification significantly enhances the structural order of the PBTTT backbone while improving thermal–mechanical properties, strengthening intrachain interactions. Comparative studies of F_6_TCNNQ-doped PBTTT-C_12_ and PBTTT-^8^O reveal that the polar n-C_7_OC_4_ side chains facilitate dopant dispersion, with F_6_TCNNQ molecules preferentially aligning within the disordered side-chain regions rather than disrupting backbone packing, as illustrated in Fig. 11b. These synergistic effects improved structural order, optimized dopant distribution, and enhanced charge transport, achieving a PF of 2.9 mW m^−1^ K^−2^. Their further investigations into side-chain engineering [64] demonstrated that the position of oxygen atoms critically influences crystallinity: when placed farther from the backbone, oxygen disrupts chain packing less, promoting order by favoring a gauche conformation.

A comprehensive mechanistic study on the side-chain electronic modulation of PBTTT polymers conducted by Kurosawa et al. [65] reveals that alkoxy substituents induce a hypsochromic shift in HOMO energies. They demonstrated the strong electron-donating effect of alkoxy groups significantly upshifts the HOMO level, resulting in a lower ionization potential. The enhanced electron-donating character induces greater backbone planarity in PBTTT, as evidenced by a higher torsional energy barrier relative to alkyl-substituted chains, which effectively reduces conformational disorder along the polymer backbone (Fig. 11c). These findings highlight the critical role of side-chain engineering in optimizing conjugated polymer performance.

Generally speaking, side-chain polarity, length, and branching profoundly impact backbone planarity, interchain π-π stacking distance/donor–acceptor overlap, and energetic disorder. The future lies in integrated computational and AI-guided precision side-chain design, exemplified by exploring synergistic functional group combinations, implementing tailored molecular asymmetry, integrating ionic functionalities for mixed conduction or controlled doping, and harnessing programmable supramolecular interactions. Crucially, AI-enabled synergistic optimization with the conjugated backbone and dopants will be the decisive factor. Achieving ultra-stable, high-performance organic TE materials through intelligent side-chain engineering presents a vital pathway toward energy harvesting and cooling applications.

#### Melting-Induced Crystallization

PBTTT demonstrates a unique advantage over conventional conductive polymers due to the presence of liquid-crystalline phases [129, 130]. These liquid crystal phases, particularly the smectic mesophase, facilitate the long-range ordering of PBTTT molecular chains, thereby significantly enhancing its TE properties. While molten heating can induce the formation of these liquid-crystalline phases in PBTTT, achieving optimal alignment requires precise control over both temperature and cooling rate. Specifically, thermal processing conditions must be carefully tailored to harness the full potential of PBTTT's liquid-crystalline behavior for improved charge transport performance.

**Fig. 12:**
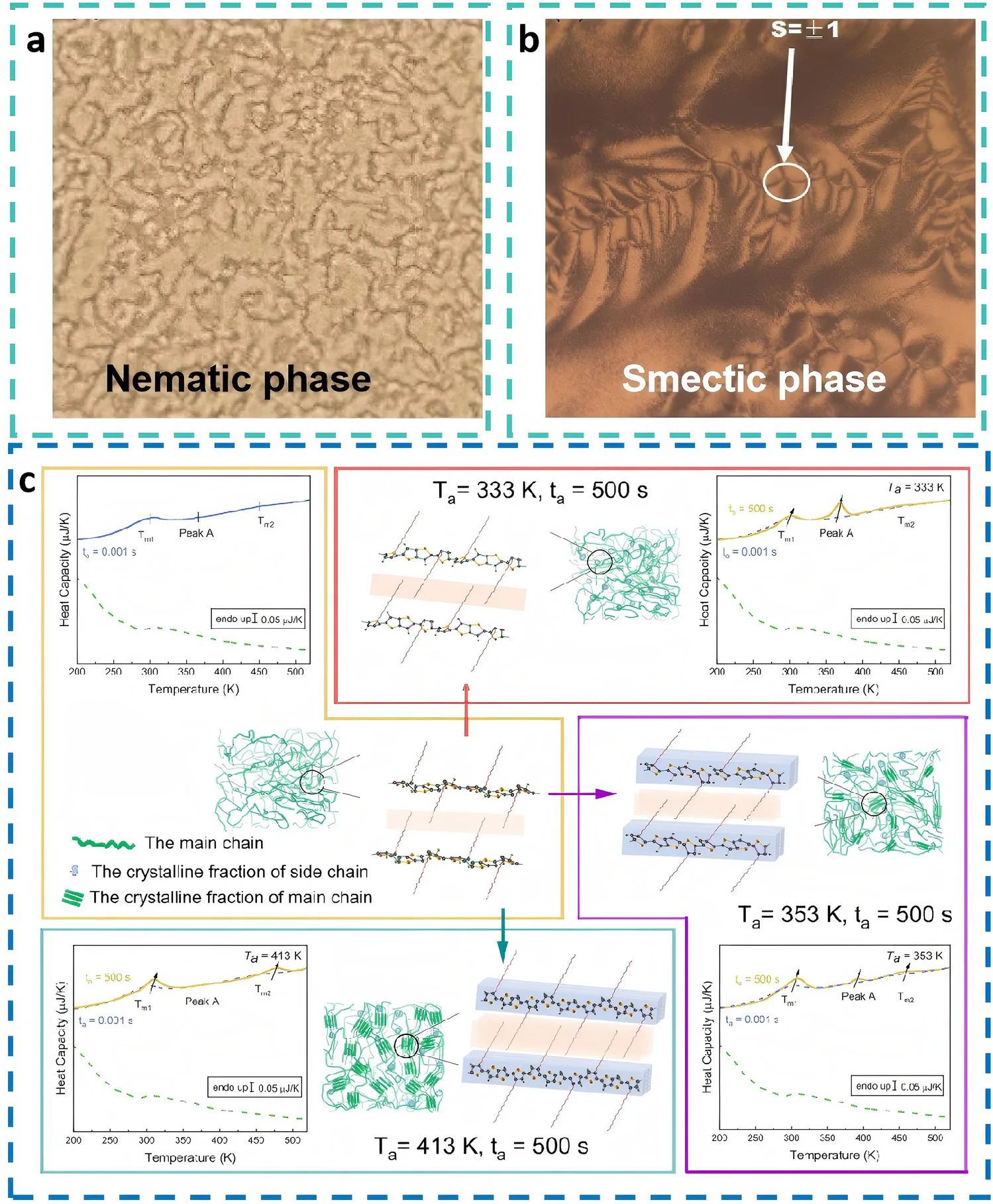
Evolution of the liquid-crystalline phase in PBTTT. **a** Optical image of PBTTT film at the nematic phase [129]. **b** Optical image of PBTTT film at the smectic phase [129]. **c** Schematic illustration of the possible molecular ordering variety involved in the conformational evolution of PBTTT at different crystallization temperatures [130]

Studies indicate that the cooling rate significantly influences the induction of two liquid crystal phases [129]. During the melt-cooling process, PBTTT can develop either a nematic phase (LC I, Fig. 12a), characterized by long-range orientational order of molecular chains without positional regularity, or a smectic phase (LC II, Fig. 12b), in which molecules form well-defined layers with both orientational and in-plane positional order. The rapid cooling of the liquid-crystalline polymer PBTTT effectively suppresses the nematic-to-smectic phase transition, significantly enhancing crystallinity and molecular ordering, leading to improved optoelectronic performance.

There remain divergent views regarding the evolution of PBTTT melt crystallization processes in current research. Zhou and coworkers [130] employed in situ Raman spectroscopy coupled with fast scanning calorimetry (FSC) to study the crystallization process of PBTTT. They found a three-stage transformation process, as shown in Fig. 12c. At low temperatures (333 K), torsional dynamics of thienothiophene and thiophene rings generated a thermotropic mesophase with local order but long-range disorder; at intermediate temperatures (353–363 K), competition between backbone crystallization and mesophase preservation resulted in imperfect crystalline domains; while at elevated temperatures (413 K), enhanced backbone and side-chain mobility facilitated direct formation of highly crystalline structures. Martin et al. [131] believed that the conventionally labeled “annealing temperature” corresponds to a transition into a liquid-crystalline state rather than a crystalline phase. They found three distinct regimes (1) Below 120 °C, crystalline melting occurs, yielding either a glassy state or supercooled liquid; (2) Between 120 and 250 °C, a smectic liquid-crystalline phase with lamellar ordering emerges, albeit with limited long-range periodicity; and (3) Above 250 °C, the polymer transitions to a fully disordered isotropic liquid.

### Recent-Developed Special Advances in PBTTT-Based TE Materials

Despite inherently lower TE performance compared to inorganic materials, state-of-the-art PBTTT-based TE materials have achieved comparable performance metrics through optimized doping techniques and crystallinity engineering. Crucially, these improvements preserve key organic advantages, including mechanical compliance and solution processability.

#### Doping Through Proton-Coupled Electron Transfer (PCET)

**Fig. 13:**
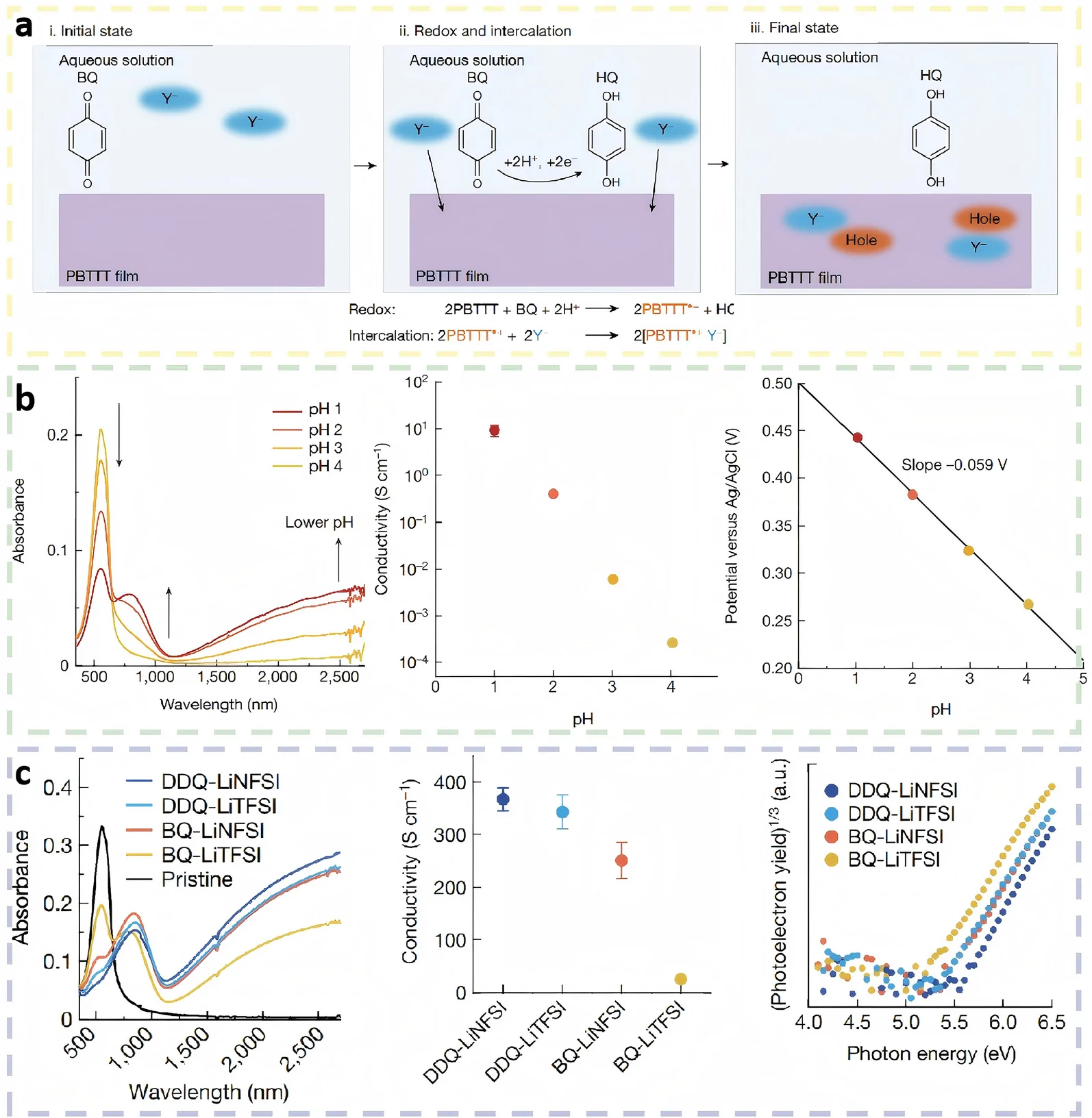
Illustration of PCET method and its experimental data. **a** Schematics of the PCET doping process with the possible reaction equations. **b** Influence of the pH of the doping levels in PBTTT film on spectral absorbance, *σ*, and electrical potential. **c** Influence of the oxidants and dopant anions on spectral absorbance, *σ* and photoelectron yield spectroscopy [132]

Yamashita et al. [132] proposed a proton-coupled electron transfer (PCET)-driven doping strategy to achieve atomic-level precision (± 25 meV) in Fermi-level engineering for PBTTT. While PCET mechanisms are ubiquitous in biological redox systems, their adaptation to organic semiconductors circumvents a critical limitation: Conventional doping inherently produces unstable dopant ions, whereas PCET synergizes non-ionic charge transfer with hydrophobic ion intercalation to decouple electrochemical activation from parasitic side reactions. As illustrated in Fig. 13a, the benzoquinone/hydroquinone (BQ/HQ) couple governs dual proton-electron transfers, with its pH-dependent redox potential self-regulated within the electrochemical stability window of aqueous environments, a design principle that inherently suppresses moisture-triggered degradation. Quantitative correlations between proton activity, optoelectronic properties, and energy-level realignment are systematically mapped in Fig. 13b. Higher proton activity (lower pH values) provides stronger oxidative power, driving the oxidation of more PBTTT molecules, thereby generating more hole carriers and significantly enhancing electrical conductivity. Meanwhile, the trend in the energy-level of PBTTT shifting with pH confirms that changes in proton activity directly determine the magnitude of the electrochemical driving force provided by the reaction. This enables precise and wide-range control over the doping degree of the semiconductor, ultimately allowing accurate setting of its Fermi level.

Beyond proton activity, the oxidizer and anion selection significantly impact charge transport efficiency. As shown in Fig. 13c, they compared the effects of two oxidizers, 2,3-dichloro-5,6-dicyano-1,4-benzoquinone (DDQ) and DQ, and two anions, bis(nonafluorobutanesulfonyl)imide (NFSI⁻) and TFSI⁻. The strong oxidizer DDQ effectively oxidizes PBTTT, injecting a higher density of hole carriers. Concurrently, the NFSI⁻ anion demonstrates exceptional charge transport properties. Consequently, the synergistic combination of the optimal oxidizer (DDQ) and the highest-conductivity anion (NFSI⁻) achieves a high electrical conductivity of 400 S cm^−1^.

#### Nanoconfined Electrochemical Ion Implantation (NEII) Doping

**Fig. 14:**
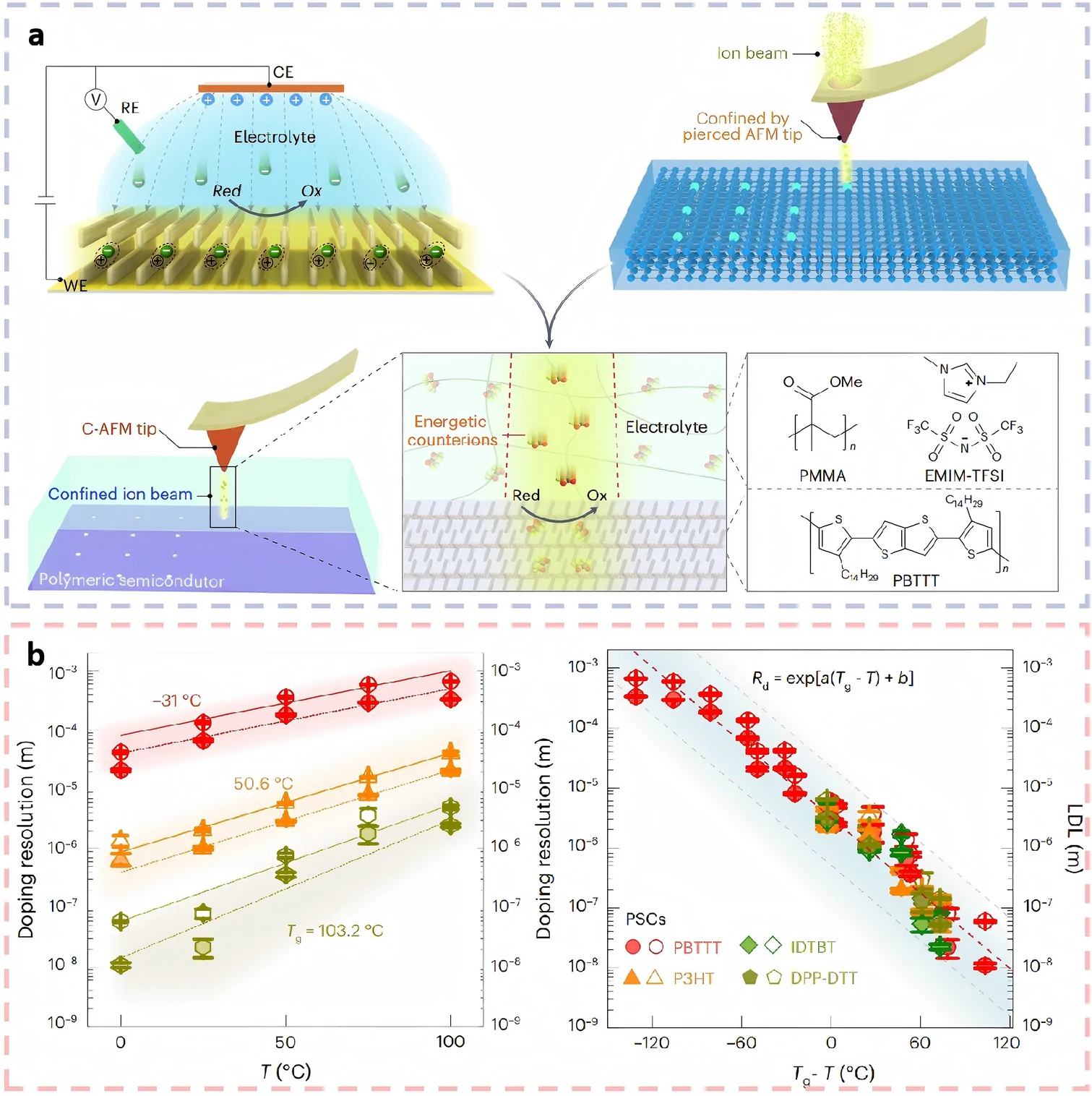
Illustration of NEII doping and its experimental data. **a** Schematics of the NEII doping process, in which the counterions is confined by both the conductive AFM tip and the reshaped fringing field in the electrolyte. **b** T- and Tg-dependent doping resolution [133]

Previous studies primarily focused on precise control of doping effects, whereas Di et al. [133] extended this capability to the spatial domain. Drawing inspiration from ion implantation techniques in inorganic semiconductors, they developed the nanoconfined electrochemical ion implantation (NEII) method, enabling sub-100-nm spatial precision doping. Figure 14a schematically illustrates the NEII mechanism: An AFM tip serves as a counter-electrode, while an electrolyte creates a fringing field that spatially constrains ion migration. This design effectively confines doping to the nanoscale. The electrolyte consists of polymethylmethacrylate (PMMA) blended with an ionic liquid, where the PMMA-to-ionic liquid ratio governs the glass transition temperature, a key parameter determining the confinement characteristics. Figure 14b systematically analyzes the effects of both temperature and Tg on doping resolution. The results demonstrate an inverse relationship: increasing Tg enhances resolution, while higher temperatures degrade it. This tunable behavior provides a powerful means to optimize spatial control in organic semiconductor doping.

Considering that doping in crystalline regions primarily enhances *σ* while doping in amorphous regions boosts the *S*, the integration of the NEII method with in situ molecular chain conformation characterization enables precise monitoring and manipulation of doping-induced structural reorganization in both ordered and disordered domains. This spatial-resolved control facilitates synergistic optimization of TE parameters, achieving maximization of PF.

#### Multi-heterojunctioned PBTTT Composite

**Fig. 15:**
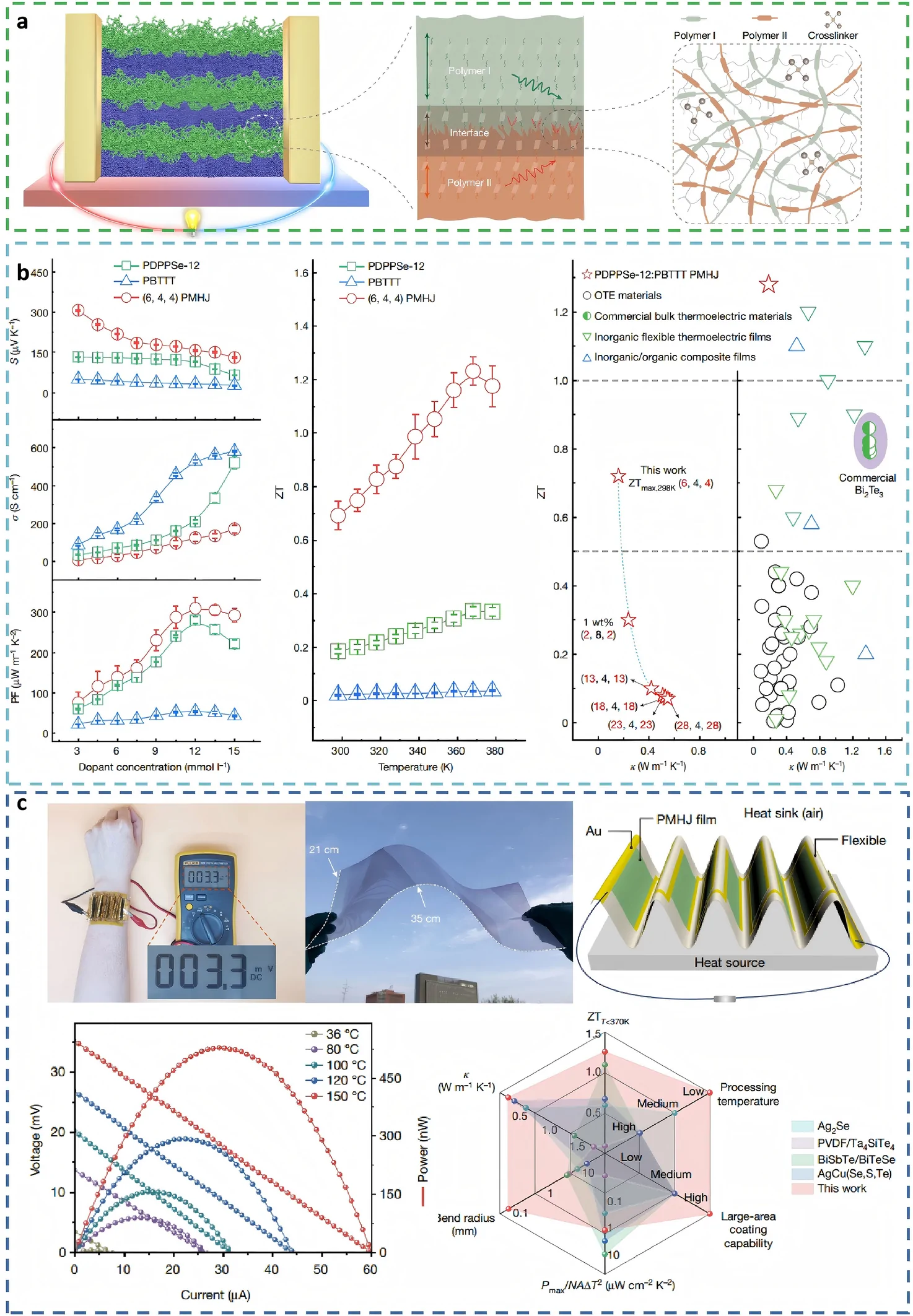
Illustration of the structure and experimental data for the multi-heterojunctioned PBTTT composite. **a** Schematic of the structure. **b** Variation of TE performance with temperature and dopant concentration, including *σ*, *S*, PF, *κ*, and *ZT*. **c** Schematic illustration of the flexible integrated TEG and power output measurement [59]

Most current strategies for the enhancement of TE properties focus overwhelmingly on electrical optimization, while systematic approaches for governing *κ* remain underdeveloped. Zhao and coworkers proposed an innovative methodology targeting *κ* suppression to optimize TE performance [59]. They designed a periodic two-dimensional heterojunction architecture, which enables precise manipulation of phonon scattering pathways, achieving a suppression of *κ* while maintaining excellent charge transport. As illustrated in Fig. 15a, the heterojunctions were constructed using selenium-substituted diketopyrrolopyrrole polymer (PDPPSe-12) and PBTTT. A four-armed azide-based crosslinker was employed to achieve nanoscale layer-by-layer stacking via ultraviolet light crosslinking. Chemical doping was implemented through immersion in FeCl_3_/nitromethane solution. This fabrication protocol exhibited excellent structural consistency, enabling large-area coating (35 × 21 cm^2^) with a uniform thickness of 16.2 ± 2.6 nm. The structure incorporates interfaces scaled to the phonon mean free path length, enabling strong interfacial phonon scattering while maintaining efficient charge transport channels. The out-of-plane *κ*_⊥_ reduced to 0.06 W m^−1^ K^−1^ (Fig. 15b), showing a substantial 55% and 76% reduction compared to pristine PBTTT and PDPPSe-12 films, respectively. The measurement of *κ*_⊥_ is mutually validated through 3*ω*-Scanning thermal microscopy (3*ω*-SThM) and the suspended microdevice method, ensuring result reliability.

Remarkably, the heterojunction simultaneously enhances both the *S* and *σ* relative to single-component films. This synergistic improvement yields an exceptional PF of 628 µW m^−1^ K^−2^ at 368 K, culminating in a record *ZT* value of 1.28. Two mechanisms underlie this performance breakthrough: (1) Doping effects that optimize carrier concentration; (2) quasi-2D charge transport in the heterostructure, where interfacial charge transfer increases electronic entropy. The flexible TE generator (TEG) architectured with the heterojunction thin-film modules (Fig. 15c) demonstrated an output power of 522 nW under an applied temperature gradient of 38 K. The TEG exhibited robust mechanical stability (> 95% conductivity retention after 100,000 bending cycles) and generated 3.3 mV open-circuit voltage on human skin. The optimized power density (1.12 μW cm^−2^ K^−2^) positions this architecture as a scalable, cost-effective platform for powering IoT devices and flexible electronics from low-grade waste heat.

### Brief Summary

Doping remains the most prevalent method for optimizing TE performance. It enhances conductivity by modulating carrier concentration, yet faces challenges such as environmental instability. While anion doping improves stability, bulky anions increase π–π stacking distance and reduce conductivity. Current doping strategies are advancing toward refinement, with progress in spatial and energy-level precision control. The PCET doping method achieves precise energy-level manipulation but is restricted by pH responsiveness, effective only in strongly acidic conditions, while rendering it unsuitable for biological environments or mildly acidic scenarios. NEII technology addresses core challenges in polymeric nanodoping through glassy electrolyte confinement effects, though it requires overcoming the “high-resolution vs. high-conductivity” trade-off dilemma.

For composites, PBTTT/CNT blends suffer from poor dispersion and phase separation, while PBTTT combined with other conductive polymers yields limited TE improvement. Composites leveraging hetero-interface phonon selective scattering can suppress lattice thermal conductivity while preserving high electrical conductivity. However, complex fabrication processes, interface thickness sensitivity, and ultrathin-film limitations result in low power output.

Regarding aggregation state control, current research primarily emphasizes crystallization mechanisms but inadequately explores harnessing crystalline control to enhance TE performance. Given that crystallinity optimization, side-chain engineering, and strategic doping collectively govern charge transport and phonon scattering dynamics, future investigations should pursue synergistic integration of these factors.

The advantages and disadvantages of the three approaches are presented in Table 2. Overall, each method possesses its own merits and limitations, while combining multiple techniques enables synergistic enhancement by leveraging their complementary effects. TE performance optimization strategies are evolving toward an integrated approach that strategically combines crystallization control, side-chain engineering, and advanced doping techniques, harnessing their complementary advantages while mitigating individual limitations. Future endeavors will leverage machine learning and other advanced techniques to conduct multivariate parameter optimization, achieving fine-tuned and customized control over TE performance.

**Table 2:** Advantages and disadvantages of doping, composites and aggregation state control

Strategy	Doping	Composites	Aggregation State Control
Mechanism	Redox reactions or ion exchange to adjust carrier concentration	Hybrids with carbon materials (CNTs, graphene) or polymers	Solvent/thermal tuning of crystallinity and chain alignment
Advantages	(1) Directly controlling carrier concentration enables precisely engineering significantly enhanced electrical conductivity; (2) Ensuring compatibility with existing device fabrication processes facilitates high-volume manufacturing	(1) Hetero-interfaces selectively scatter phonons while preserving charge carriers, holding the promise for decoupling thermal and electronic transport (2) Enabling the integration of multiple functionalities	(1) Conductivity enhanced through carrier transport trajectory optimization, without disrupting the crystal lattice structure (2) Suppression of lattice thermal conductivity via nano-boundary design; (3) Solution processability with simplified fabrication
Disadvantages	(1) Dopants exhibit susceptibility to deactivation and chemical/environmental sensitivity; (2) High doping levels readily compromise lattice integrity and disrupt crystalline structure; (3) While doping boosts *σ*, it concurrently depresses the *S* and frequently elevates *κ*	(1) PBTTT demonstrates poor compatibility with CNTs, leading to unfavorable phase separation (2) Blending with P3HT offers only moderate performance improvement, thus necessitating complementary doping strategies (3) The fabrication of heterojunction structures is complex. The thin film is very thin, resulting in low output power	(1) Slow crystallization kinetics and poor repeatability (2) Sensitivity to solvent polarity; (3) Deficient TE enhancement necessitates compensation through strategic doping

## TE Properties

In the previous section, we summarized specific methods to improve TE performance. In this section, we have compiled and summarized the reported TE of PBTTT. Table 3 gives the TE performances of PBTTT-related materials reported in literature. Below, we summarize the critical advancements in each category.

**Table 3:** TE performances of PBTTT-related materials reported in literature

Type of PBTTT	Treatment	*ZT*	PF, μW m^−1^ K^−2^	*σ*, *S* cm^−1^	*S*, μV K^−1^	*κ*, W m^−1^ K^−1^	Refs	Year
PBTTT-C_14_	Drop casting; Doped with NOPF_6_; Annealed		0.98	53.8	13.5		[134]	2012
PBTTT-C_14_	Solution casting; Solution doped with F_4_TCNQ		1.3 ± 0.4	3.51 ± 0.05	60 ± 9		[71]	2014
PBTTT-C_14_	Vapor deposition of FTS		25 ± 8	466 ± 0.1	23 ± 4		Ditto	
PBTTT-C_12_	Drop casting; Doped with TFSI^−^		3.71	220	13		[135]	2014
PBTTT-C_14_	Vapor doping with F_4_TCNQ; Annealed		32 ± 9	220.00 ± 0.02	39 ± 5		[84]	2017
PBTTT-C_14_	Vapor doping with F_2_TCNQ; Annealed		70 ± 20	36 ± 3	140 ± 20		Ditto	
PBTTT-C_12_	High-T rubbing; Subsequent solution doping with FeCl_3_		1944 ± 626 // (in-plane)	(2.2 ± 0.5) × 10^5^ //	9.4 ± 0.5 //		[75]	2019
			0.47 ⊥ (out-of-plane)	2100 ± 300 ⊥	1.5 ± 0.5 ⊥			
PBTTT-C_12_ PBTTTS-C_12_ blend	Drop casting; Solution doping with NOBF_4_		1.2	510	4.85		[136]	2019
PBTTT-C_7_OC_4_	F_6_TCNNQ doping; High-T rubbing up to 240 °C		2900	5 × 10^4^	24.1		[127]	2022
PBTTT-^8^O	Doped with F_6_TCNNQ; High-T rubbing		256	3250	4.2		[82]	2022
PBTTT-C_14_ P3HT blend	F_4_TCNQ	0.1	97	101	98	0.3	[74]	2022
PBTTT-C_14_	Ion Exchange Doped; Anion: TFSI^−^		34.2 ± 4.3	70	71		[94]	2023
PBTTT-C_14_	Ion Exchange Doped; Anion: TFSM^−^		30.9 ± 4.5	100	30.9		Ditto	
PBTTT-C_14_	Ion Exchange Doped; Anion: TFO^−^		24.2 ± 2.7	105	48		Ditto	
PBTTT-^11^O	Doped with F_6_TCNNQ and aligned by high-T rubbing		496.2	59	29		[64]	2024
PBTTT-^8^O	Ditto		2928.2	5 × 10^4^	24.2		Ditto	
PBTTT-^5^O	Ditto		1324.8	23,000	24		Ditto	
PBTTT-^3^O	Ditto		729	10,000	27		Ditto	
PBTTT-C_14_	Two step doping: Solution doping with F_4_TCNQ, followed by ion exchange doping		26.97 ± 1.05	941.1	16.93 ± 0.4		[69]	2024
PBTTT-C_12_	Ion-exchange doping; Blending of high and low molecular weight chains		173	4810	18.9		[122]	2024
PBTTT-C_14_	Doped with BCF		230	140	128		[73]	2024
PgBTTT	Doped with BCF		223 ± 4	2180 ± 360	32 ± 1.3		[87]	2024
PBTTT-C_12_	Modifying the substrate with self-assembled monolayers		30.7	320	31		[137]	2024
PBTTT -C_14_	PDPPSe-12 and PBTTT heterojunction structure	1.28	628 (at 368 K)	196	179	0.18	[59]	2024
PBTTT -C_14_	Supramolecular nucleating agent (PDA) doping strategy		176	1894	30.4		[138]	2025

### Electrical Conductivity

The *σ* is determined by both the charge carrier concentration and the carrier mobility, as expressed by the equation:

2 $$\sigma =ne\mu .$$

Here, *n* represents the carrier concentration, e is the elementary charge, and *μ* is the carrier mobility. By employing doping strategies [134–136], electron acceptors or donors can be introduced into conjugated polymers to increase carrier concentration. In addition, chain conformation modulations [137, 138] can influence charge transport properties and enhancing mobility. It has been proved that gas-phase doping can increase the PF by an order of magnitude compared to solution doping [90]. In recent years, the development of anion exchange doping has further improved doping efficiency [69, 94]. The optimization strategies discussed in the third section primarily focus on enhancing *σ*. According to Table 3, it can be seen that the *σ* of PBTTT has increased by three to four orders of magnitude from its initial value of less than 5 S cm^−1^ [71, 127].

### Seebeck Coefficient

According to the Mott formula [139] in degenerately doped semiconductors, the expression for *S* is:

3 $$S = \frac{{\pi^{2} k_{B}^{2} }}{3e}T\left\{ {\frac{{{\mathrm{d}}n\left( E \right)}}{{n{\mathrm{d}}E}} + \frac{{{\mathrm{d}}\mu \left( E \right)}}{{\mu {\mathrm{d}}E}}} \right\}_{{E = E_{{\mathrm{f}}} }} .$$

*E* represents the carrier energy, and d*n*(*E*)/d*E* is equivalent to the slope of the carrier Density of States (DOS). As shown in Eqs. (2) and (3), carrier concentration and mobility are positively correlated with *σ* but negatively correlated with *S*. Consequently, there exists a trade-off between optimizing *S* and *σ*.

Some studies are dedicated to decoupling *S* and *σ*. Katz et al. [136] found that using nitrosonium tetrafluoroborate (NOBF_4_), a kind of strong polar dopant, can decouple *S* and *σ*. Their results demonstrate that while either *S* or *σ* is increased, the other parameter can be stabilized or slightly increased through offsetting energy levels and improving intermolecular interactions. Considering that PBTTT is thermoplastic, Brinkmann et al. [64, 82, 127] improved the long-range order of PBTTT polymer chains through high-T rubbing, stretching, and annealing. The structural optimization effectively modulates charge transport pathways while minimizing the adverse impact on thermopower, thereby providing another viable pathway for partial *S*-*σ* decoupling.

In conductive polymers like PEDOT, the energy filtering strategy has been widely explored to decouple the *S* and *σ* [140, 141]. By introducing controlled energy barriers, low-energy charge carriers can be selectively filtered out, thereby increasing the average carrier energy. However, in PBTTT-based systems, comparable studies remain scarce. Despite its promising TE properties, the application of energy filtering and its impact on *S-σ* decoupling, has not yet been systematically investigated, presenting an important avenue for future research.

### Thermal Conductivity

The total *κ* consists of two components: electronic thermal conductivity (*κ*ₑ) and phononic thermal conductivity (*κ*ₚ),

4 $$\kappa ={\kappa }_{\mathrm{e}}+{\kappa }_{\mathrm{p}}.$$

For crystalline materials, *κ*_e_ and *σ* obey the Wiedemann–Franz law,

5 $$\kappa_{{\mathrm{e}}} = LT\sigma .$$

Here, L represents the Lorenz number. For TE materials, high *σ* but low *κ* is desired. Therefore, to improve TE conversion efficiency, we aim to minimize *κ*ₚ while preserving *κ*ₑ.

Currently, experimental data on the *κ* of PBTTT remains limited, primarily due to the significantly greater technical challenges associated with thermal measurements compared to electrical characterization methods. The most common measurement for *κ* is 3-*ω* method [142, 144], which determines in-plane *κ* by applying a sinusoidal alternating current (1-*ω*) to a metal microheater, inducing axial temperature oscillations via Joule heating. The 1-*ω* input signal generates a 3-*ω* voltage signal, and combined with the one-dimensional heat diffusion equation, the material’s in-plane *κ* can be derived.

The fundamental challenge in *κ* regulation resides in the inherent coupling between thermal and electrical properties. Taking inspiration from inorganic TE research, researchers engineered interfaces at specific scales by leveraging the disparity in mean free paths between electrons and phonons [59], achieving selective enhancement of phonon scattering while preserving electronic transport. Furthermore, a systematic investigation led by Campoy-Quiles et al. [109] on 17 conjugated polymers with varying chain conformations revealed that molecular chain conformation critically affects the *κ–σ* decoupling. They found that for structures with long-range order, *κ* and *σ* are positively correlated and cannot be decoupled. For amorphous and short-range ordered conjugated polymers, effective decoupling of charge and thermal transport can be achieved. Their research findings provide a research direction for enhancing TE performance by controlling *κ*. In addition, introducing interfaces in composite systems can also effectively reduce *κ*.

## Summary and Outlook

### Summary of Research Status

Positioned as a uniquely processable material in OTE field, PBTTT has demonstrated dual-solvent/melt-processing adaptability, thus occupying a pivotal position among conducting polymers for energy harvesting applications. This review systematically examines the cutting-edge progress in PBTTT-based TE materials, with particular emphasis on synthesis strategies, performance enhancement mechanisms, and emerging technological frontiers.

Regarding synthesis protocols, PBTTT and its derivatives primarily employ the Stille cross-coupling methodology for backbone construction. Subsequent solution-processing techniques (e.g., spin coating, blade coating, floating film transfer) dominate composite fabrication, endowing PBTTT architectures with tailorable nanomorphology and device compatibility.

Critical advancements in optimizing TE performance primarily focus on three strategic approaches: (1) Charge carrier engineering via doping methodologies, including solution-phase doping, vapor-phase infiltration, and anion exchange doping; (2) Composite structure regulation, where synergistic combinations of material systems increasingly outperform single-component modifications; (3) Aggregation state control, which can be achieved through solvent optimization, thermal processing, and molecular chain design. Significantly, singular enhancement strategies have become inadequate to address contemporary performance requirements. To maximize TE property enhancements, state-of-the-art research emphasizes the synergistic integration of multiple approaches. For instance, combining doping with high-T rubbing, backbone design with anion exchange, and solid-state diffusion with anion exchange.

Furthermore, we have summarized the existing literature on the TE data of PBTTT-related materials. The most extensively studied TE properties include *S* and *σ*. There exists a trade-off relationship between *S* and *σ*, and researchers are dedicated to decoupling the mutual influence between the two, aiming to maximize the PF. Over the course of a decade, the PF of PBTTT has increased from initially being less than 1 μW m^−1^ K^−2^ by two to three orders of magnitude.

Finally, we present the latest research developments regarding PBTTT in the field of TE. PCET doping not only enhances the stability of dopants in the environment but also allows for precise Fermi level tuning with an accuracy of 25 meV. NEII doping enables spatially high-resolution doping, with resolutions as low as 100 nm. Multi-heterojunctioned PBTTT composites simultaneously regulate *σ*, *S*, and *κ*, achieving a remarkable *ZT* value of up to 1.28 at 368 K.

### Challenges and Opportunities

**Fig. 16:**
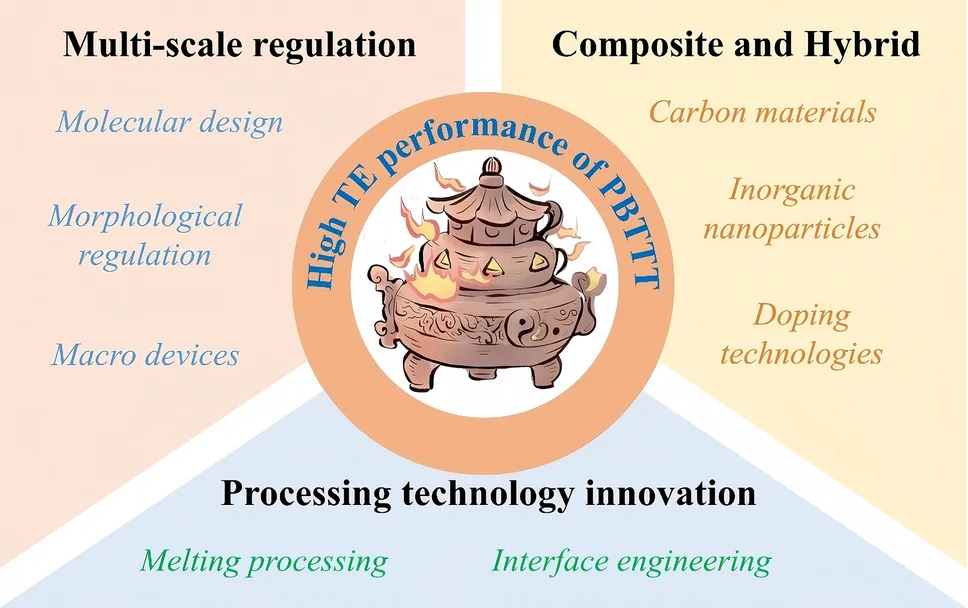
Potential strategies for achieving high TE performance with PBTTT

Although significant progress has been made in improving the TE performance of PBTTT through various optimization strategies in recent years, further research has unveiled emerging opportunities and challenges. As illustrated in Fig. 16, these include but are not limited to the following three key aspects:

(1) Multiscale regulation. Multiscale regulation enables synergistic optimization of TE performance, encompassing molecular-scale design, micro/nanomorphological control, and macroscopic device engineering. At the molecular level, backbone optimization and side-chain engineering of PBTTT can be employed to modify conjugation lengths and band structures through chemical modifications, thereby altering charge transport mechanisms. The micro/nanostructural level focuses on regulating the liquid-crystalline phase ordering of PBTTT. Precise control over crystallinity and phase separation is critical for enhancing carrier mobility while suppressing *κ*. At the macroscopic device level, despite PBTTT has achieved a notable *ZT* value of 1.28, its power output still remains limited to 522 nW. The low output power primarily stems from its intrinsic resistance and the high contact resistance during device assembly. Therefore, strengthening innovation at the macro device level, such as jointless p-n and device integration design, can improve overall TE performance. This discrepancy highlights the need for holistic optimization across all length scales to bridge the gap between material-level properties and device performance, and significant to improve energy conversion efficiency.

(2) Composite and hybrid strategies. Many prominent studies have demonstrated the significant impact of composite hybridization approaches on enhancing the TE performance of PBTTT, including those carbon material incorporation, inorganic nanoparticle dispersion, and doping. Carbon-based hybridization facilitates the formation of continuous conductive pathways within the polymer matrix, dramatically improving charge carrier mobility. Graphene and carbon nanotubes have proven particularly effective in establishing percolation networks that enhance *σ* while maintaining favorable *S*. Inorganic nanoparticle integration leverages interfacial effects to promote energy filtering mechanisms. In addition, new doping methods also have a surprising effect on improving TE performance. This selective scattering process preferentially transmits high-energy charge carriers, thereby simultaneously increasing the *S* and preserving *σ*, which is a crucial advantage for achieving high *ZT* values. However, the research on the *κ* of PBTTT is currently insufficient. The *κ* of conjugated polymers encompasses lattice thermal conduction and electronic thermal conduction. In metallic materials, the *κ*_e_ and *σ* directly adhere to the Wiedemann–Franz Law. However, in conjugated polymers, whether this law holds true remains an opening question. Therefore, only by investing more in fundamental understanding of the mechanisms governing *κ* in PBTTT can we effectively reconcile the interrelationships among these three critical parameters to ultimately maximize the *ZT* value. This requires accurate thermal measurements, an area where traditional techniques like time-domain thermoreflectance (TDTR) and the 3-*ω* method face limitations due complex sample preparations, high instrumentation costs, substrate interference effects and so on. To overcome these constraints in organic TE film characterization, Wang et al. [145] recently proposed a laser spot periodic heating technique based on sub-region phase fitting. This approach minimizes sample preparation requirements and enables rapid determination of thermal conductivity, promising significant advances for future thermal measurements of PBTTT and other organic TE films.

(3) Innovative material processing techniques. Currently, the advantages of melt processibility for PBTTT have not been fully utilized. While most conjugated polymers are typically processed using solution-based methods to produce TE films, PBTTT can be processed not only via solution methods but also through melt-processing techniques such as injection molding, extrusion, rolling, melt spinning, and others. However, there is currently limited research on using melt-processing methods to fabricate PBTTT-based TE materials. Only a small amount of literature reports on the use of high-T rubbing to process PBTTT films, aiming to improve molecular chain orientation and enhance TE performance. The impact of melt-processing methods on its TE properties requires further investigation. Additionally, interfacial engineering during processing, such as modulating energy levels, microstructures, and phonon/electron transport, should be considered to optimize TE performance.

Anyway, of the individual strategies, improvements are often limited to specific aspects of performance. For instance, doping generally yields the most immediate boost in *σ*, whereas composite engineering can tackle *κ* via phonon scattering, and morphology control often helps balance *σ* and *S*. Extensive evidence indicates that the synergistic integration of methods (such as first orienting polymer chains before doping, or designing new main chains that better accommodate dopants) is the key to achieving record-breaking performance. Only through close coordination among diverse strategies can the advancement of PBTTT applications be accelerated.
